# Deep Supervised, but Not Unsupervised, Models May Explain IT Cortical Representation

**DOI:** 10.1371/journal.pcbi.1003915

**Published:** 2014-11-06

**Authors:** Seyed-Mahdi Khaligh-Razavi, Nikolaus Kriegeskorte

**Affiliations:** Medical Research Council, Cognition and Brain Sciences Unit, Cambridge, United Kingdom; University College London, United Kingdom

## Abstract

Inferior temporal (IT) cortex in human and nonhuman primates serves visual object recognition. Computational object-vision models, although continually improving, do not yet reach human performance. It is unclear to what extent the internal representations of computational models can explain the IT representation. Here we investigate a wide range of computational model representations (37 in total), testing their categorization performance and their ability to account for the IT representational geometry. The models include well-known neuroscientific object-recognition models (e.g. HMAX, VisNet) along with several models from computer vision (e.g. SIFT, GIST, self-similarity features, and a deep convolutional neural network). We compared the representational dissimilarity matrices (RDMs) of the model representations with the RDMs obtained from human IT (measured with fMRI) and monkey IT (measured with cell recording) for the same set of stimuli (not used in training the models). Better performing models were more similar to IT in that they showed greater clustering of representational patterns by category. In addition, better performing models also more strongly resembled IT in terms of their within-category representational dissimilarities. Representational geometries were significantly correlated between IT and many of the models. However, the categorical clustering observed in IT was largely unexplained by the unsupervised models. The deep convolutional network, which was trained by supervision with over a million category-labeled images, reached the highest categorization performance and also best explained IT, although it did not fully explain the IT data. Combining the features of this model with appropriate weights and adding linear combinations that maximize the margin between animate and inanimate objects and between faces and other objects yielded a representation that fully explained our IT data. Overall, our results suggest that explaining IT requires computational features trained through supervised learning to emphasize the behaviorally important categorical divisions prominently reflected in IT.

## Author Summary

Computers cannot yet recognize objects as well as humans can. Computer vision might learn from biological vision. However, neuroscience has yet to explain how brains recognize objects and must draw from computer vision for initial computational models. To make progress with this chicken-and-egg problem, we compared 37 computational model representations to representations in biological brains. The more similar a model representation was to the high-level visual brain representation, the better the model performed at object categorization. Most models did not come close to explaining the brain representation, because they missed categorical distinctions between animates and inanimates and between faces and other objects, which are prominent in primate brains. A deep neural network model that was trained by supervision with over a million category-labeled images and represents the state of the art in computer vision came closest to explaining the brain representation. Our brains appear to impose upon the visual input certain categorical divisions that are important for successful behavior. Brains might learn these divisions through evolution and individual experience. Computer vision similarly requires learning with many labeled images so as to emphasize the right categorical divisions.

## Introduction

Visual object recognition is thought to rely on a high-level representation in the inferior temporal (IT) cortex, which has been intensively studied in humans and monkeys [1]–[12]. Object images that are less distinct in the IT representation are perceived as more similar by humans [10] and are more frequently confused by humans [13] and monkeys [6]. IT cortex represents object images by response patterns that cluster according to conventional categories [6], [7], [9], [14]–[16]. The strongest categorical division appears to be that between animates and inanimates. Within the animates, faces and bodies form separate sub-clusters [6], [7], [15].

Previous studies have compared the representational dissimilarity matrices (RDMs) of a small number of models (mainly low-level models) with human IT and some other brain areas [7], [17]–[19]. One of the previously tested models was the HMAX model [20], [21], which was designed as a model of IT taking many of its architectural parameters from the neuroscience literature. The internal representation of one variant of the HMAX model failed to fully explain the IT representational geometry [7]. In particular, the HMAX model did not account for the category clustering observed in the IT representation.

This raises the question if any existing computational vision models, whether motivated by engineering or neuroscientific objectives, can more fully explain the IT representation and account for the IT category clustering. IT clearly represents visual shape. However, the degree to which categorical divisions and semantic dimensions are also represented is a matter of debate [22], [23]. If visual features constructed without any knowledge of either category boundaries or semantic dimensions reproduced the categorical clusters, then we might think of IT as a *purely* visual representation. To the extent that knowledge of categorical boundaries or semantic dimensions is required to build an IT-like representation, IT is better conceptualized as a visuo-semantic representation.

Here we investigate a wide range of computational models [24] and assess their ability to account for the representational geometry of primate IT. Our study addresses the question of how well computational models from computer vision and neuroscience can explain the IT representational geometry. In particular, we investigated whether models not specifically optimized to distinguish categories can explain IT's categorical clusters and whether models trained using supervised learning with category labels better explain the IT representational geometry.

Evaluating a computational model requires a framework for relating brain representations and model representations. One approach is to directly predict the brain responses to a set of stimuli by means of the computational models. Because of its roots in the computational neuroscience of early visual areas, this approach is often referred to as receptive-field modeling. It has been successfully applied to cell recording, e.g. [25], and fMRI data, e.g. [26]–[28]. Here we attempt to test complex network models whose internal representations comprise many units (ranging from 99 to 2,904,000). The brain-activity data consist of hundreds of measured brain responses. In this scenario, the linear correspondency mapping between model units and brain responses is complex (a matrix of number of model units by number of brain responses). Estimating this linear map is statistically costly, requiring a combination of substantial additional data (for a separate set of stimuli) and prior assumptions (for regularizing the fit). Here we avoid these complications by testing the models in the framework of representational similarity analysis (RSA) [17], [18], [29], [30], in which brain and model representations are compared at the level of the dissimilarity structure of the response patterns. The models, thus, predict the dissimilarities among the stimuli in the brain representation. This approach relies on the assumption that the measured responses preserve the geometry of the neuronal representational space. The representational geometry would be conserved to high precision if the measured responses sampled random dimensions of the neuronal representational space [31], [32]. The RSA framework enables us to test any pre-trained model directly with data from a single stimulus set.

We tested a total of 37 computational model representations. Some of the models mimic the structure of the ventral visual pathway (e.g. HMAX, VisNet, Stable model, SLF) [20], [21], [33]–[37]; others are more broadly biologically motivated (e.g. Biotransform, convolutional network) [38]–[41]; and the others are well-known computer-vision models (e.g. GIST, SIFT, PHOG, PHOW, self-similarity features, geometric blur) [42]–[48]. Some of the models use features constructed by engineers without training with natural images (e.g. GIST, SIFT, PHOG). Others were trained in an unsupervised fashion (e.g. HMAX and VisNet).

We also tested models that were supervised with category labels. Two of the models (GMAX and supervised HMAX) [35] were trained in a supervised fashion to distinguish animates from inanimates, using 884 training images. In addition, we tested a deep supervised convolutional neural network [41], trained by supervision with over a million category-labeled images from ImageNet [49].

We also attempted to recombine model features, so as to construct a representation resembling IT in both its categorical divisions and within-category representational geometry. We linearly recombined the features in two ways: (a) by *reweighting* features (thus stretching and squeezing the representational space along its original axes) and (b) by *remixing* the features, creating new features as linear combinations of the original features (thus performing general affine transformations). All unsupervised and supervised training and all reweighting and remixing was based on sets of images nonoverlapping with the image set used to assess how well models accounted for IT.

We analyzed brain responses in monkey IT (mIT; cell recording data acquired by Kiani and colleagues [6]) and human IT (hIT; fMRI data from [7]) for a rich set of color images of isolated objects spanning multiple animate and inanimate categories. The human fMRI measurements covered the entire ventral stream, so we also tested the models on fMRI data from the foveal confluence of early visual cortex (EVC), the lateral occipital complex (LOC), the fusiform face area (FFA), and the parahippocampal place area (PPA).

Internal representations of the HMAX model (the C2 stage) and several computer-vision models performed well on EVC. Most of the models captured some component of the representational dissimilarity structure in IT and other visual regions. Several models clustered the human faces, which were mostly frontal and had a high amount of visual similarity. However, all the unsupervised models failed to cluster human and animal faces that were very different in visual appearance in a single face cluster, as seen for human and monkey IT. The unsupervised models also failed to replicate IT's clear animate/inanimate division. The deep supervised convolutional network better captured the categorical divisions, but did not fully replicate the categorical clustering observed in IT. We proceeded to remix the features of the deep supervised model to emphasize the major categorical divisions of IT using maximum-margin linear discriminants. In order to construct a representation resembling IT, we combined these discriminants with the different representational stages of the deep network, weighting each discriminant and layer of the deep network so as to best explain the IT representational geometry. The resulting IT-geometry model, when tested with crossvalidation to avoid overfitting to the image set, explains our IT data. Our results suggest that intensive supervised training with large sets of labeled images might be necessary to model the IT representational space.

## Results

**Figure 1:**
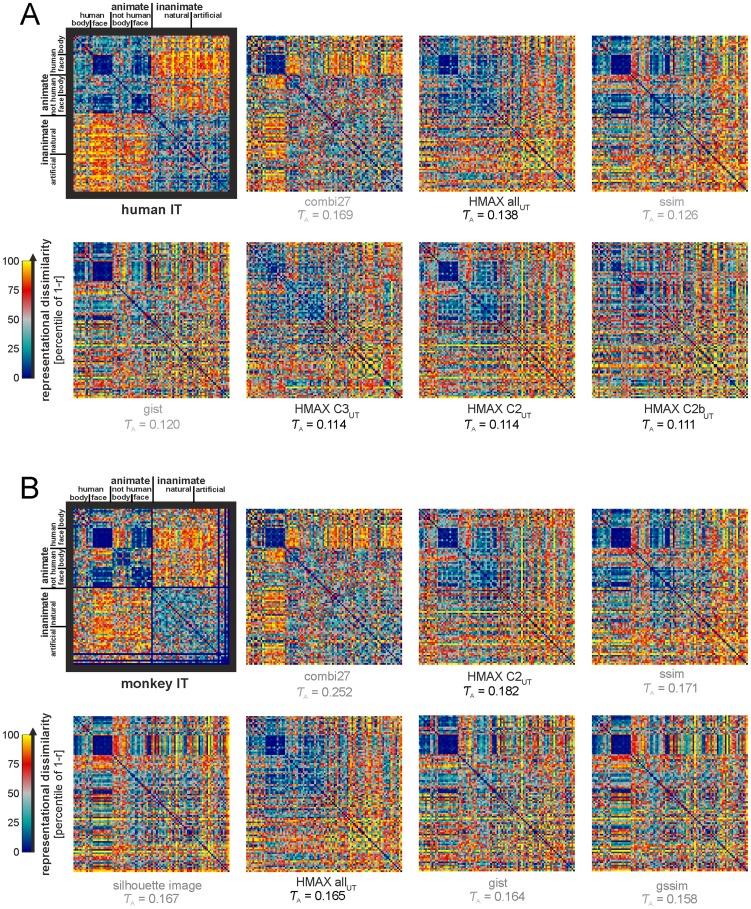
Representational dissimilarity matrices for IT and for the seven best-fitting not-strongly-supervised models. The IT RDMs (black frames) for human (**A**) and monkey (**B**) and the seven most highly correlated model RDMs (excluding the representations in the strongly supervised deep convolutional network). The model RDMs are ordered from left to right and top to bottom by their correlation with the respective IT RDM. These are the seven most higly correlated RDMs among the 27 models that were not strongly supervised and their combination model (combi27). Biologically motivated models are in black, computer-vision models are in gray. The number below each RDM is the Kendall τ_A_ correlation coefficient between the model RDM and the respective IT RDM. All correlations are statistically significant. For statistical inference, see Figure 2. For model abbreviations and RDM-correlation p values, see Table 1. For other brain ROIs (i.e. LOC, PPA, FFA, EVC) see Figure S1 and Table 1. The RDMs here are 96×96, including the four stimuli we did not have monkey data for. The corresponding rows and columns are shown in blue in the mIT RDM and were ignored in the RDM comparisons.

**Figure 2:**
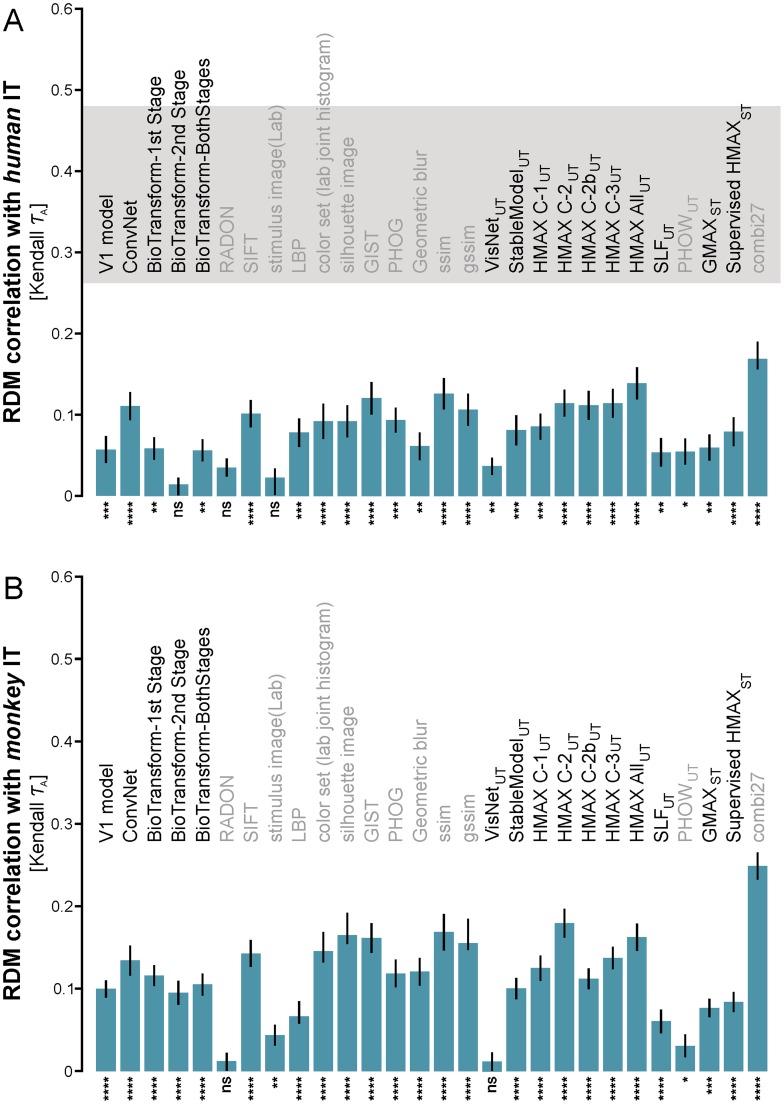
The not-strongly-supervised models fail to fully explain the IT data. The bars show the Kendall-*τ*_A_ RDM correlations between the not-strongly-supervised models and IT for human (**A**) and monkey (**B**). The error bars are standard errors of the mean estimated by bootstrap resampling of the stimuli. Asterisks indicate significant RDM correlations (random permutation test based on 10,000 randomizations of the stimulus labels; ns: not significant, p<0.05: *, p<0.01: **, p<0.001: ***, p<0.0001: ****). Most models explain a small, but significant portion of the variance of the IT representational geometry. The noise ceiling (gray bar) indicates the expected correlation of the true model (given the noise in the data). The upper and lower edges of the gray horizontal bar are upper and lower bound estimates of the maximum correlation any model can achieve given the noise. None of the not-strongly-supervised models reaches the noise ceiling. The noise ceiling could not be estimated for mIT, because the available data were from only two animals. Models with the subscript ‘UT’ are *unsupervised trained* models, models with the subscript ‘ST’ are *supervised trained* models, and others without a subscript are untrained models. Note that the supervised models included here were “weakly supervised”, i.e. with small numbers (884) of category-labeled images. Biologically motivated models are set in black font, and computer-vision models are set in gray font.

**Figure 3:**
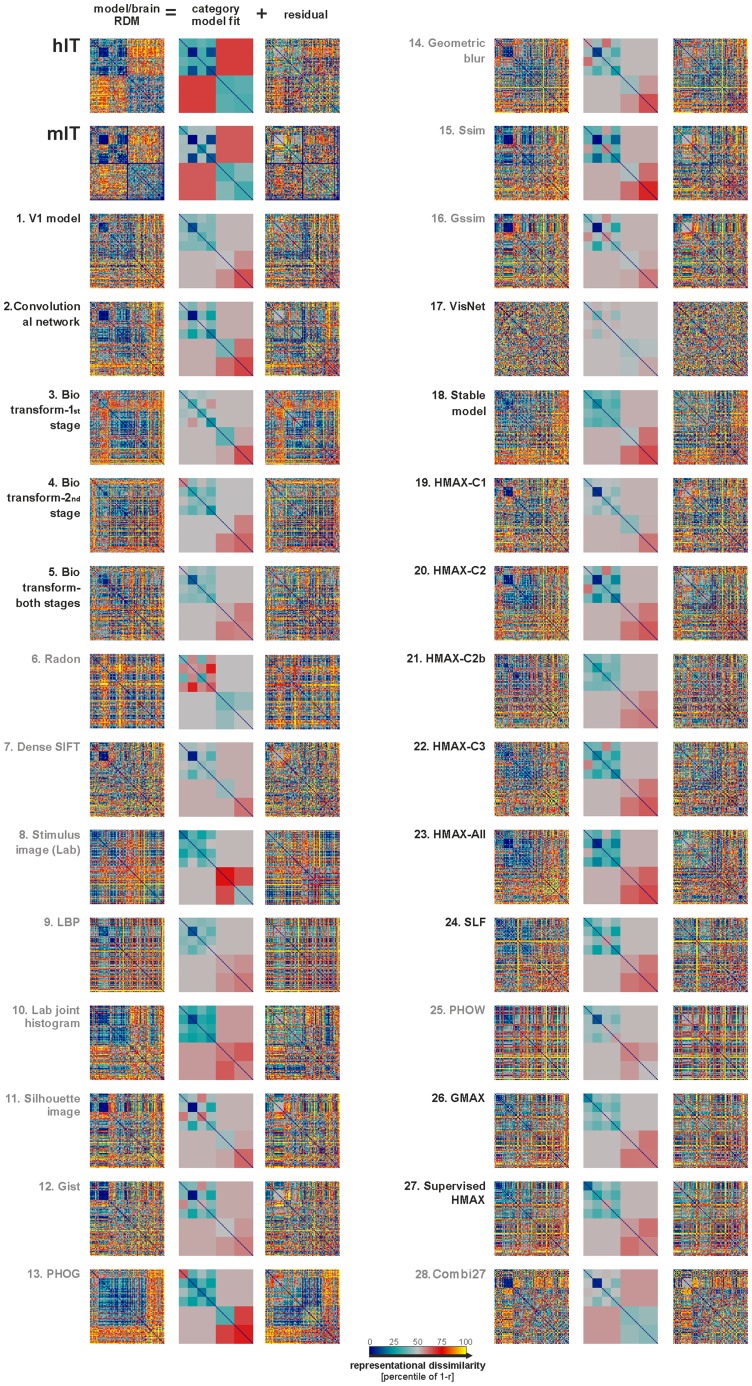
IT-like categorical structure is not apparent in any of the not-strongly-supervised models. Brain and model RDMs are shown in the left columns of each panel. We used a linear combination of category-cluster RDMs (Figure S5) to model the categorical structure (least-squares fit). The categories modeled were animate, inanimate, face, human face, non-human face, body, human body, non-human body, natural inanimates, and artificial inanimates. The fitted linear-combination of category-cluster RDMs is shown in the middle columns. This descriptive visualization shows to what extent different categorical divisions are prominent in each RDM. The residual RDMs of the fits are shown in the right column. For statistical inference, see Figure 4.

**Figure 4:**
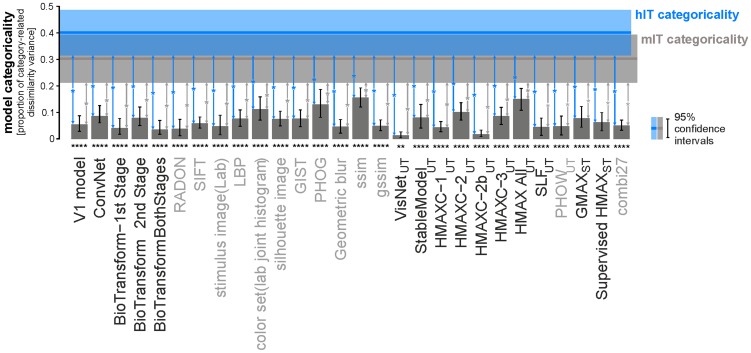
The not-strongly-supervised models are less categorical than IT. Categoricality was measured using a categoricality index (vertical axis) for each model and brain RDM. The categoricality index is defined as the proportion of RDM variance explained by the category-cluster model (Figure S5), i.e. the squared correlation between the fitted category-cluster model and the RDM it is fitted to. Bars show the categoricality index for each of the not-strongly-supervised models. The blue (gray) line shows the categoricality index for hIT (mIT). Error bars show 95%-confidence intervals of the categoricality index estimates for the models. The 95%-confidence intervals for hIT and mIT are shown by the blue and gray shaded regions, respectively. Significant categoricality indices are marked by stars underneath the bars (* p<0.05, ** p<0.01, *** p<0.001, **** p<0.0001). Error bars are based on bootstrapping of the stimulus set, and the p-values are obtained by category label randomization test. Significant differences between the categoricality indices of each model and hIT (inference by bootstrap resampling of the stimuli) are indicated by blue vertical arrows (p<0.05, Bonferroni-adjusted for 28 tests). The corresponding inferential comparisons for mIT are indicated by gray vertical arrows. Categoricality is significantly greater in hIT and mIT than in any of the 28 models. This analysis is based on equating the noise level in the models with that of hIT (Materials and Methods). Similar results obtain for a conservative inferential analysis comparing the categoricality of the noise-less models with that of the noisy estimates for hIT and mIT (Figure S9).

**Figure 5:**
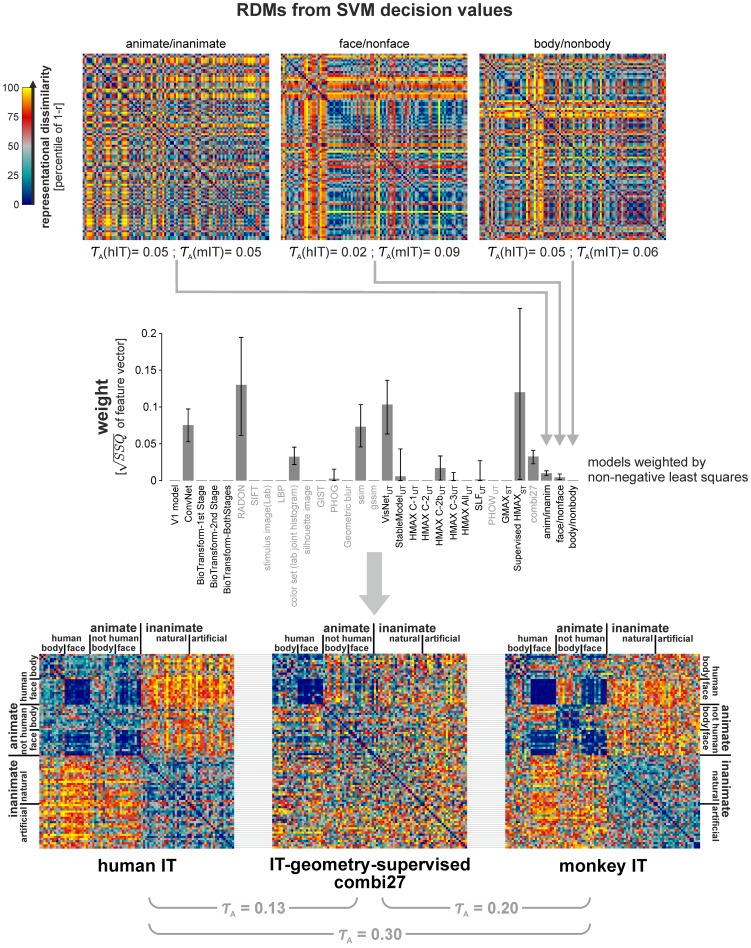
Remixing and reweighting features of the not-strongly supervised models does not explain IT. In order to build an IT-like representation, we attempted to remix the features to strengthen relevant categorical divisions. We trained three linear SVM classifiers (for animate/inanimate, face/nonface, and body/nonbody) on the combi27 features using 884 training images (separate from the set we had brain data for). RDMs for the resulting SVM decision values for the 92 images presented to humans and monkeys are shown at the top. The Kendall-*τ*_A_ RDM correlations with hIT and mIT are stated underneath the RDMs. The RDM correlations are low, but all three are statistically significant (p<0.05). We further attempted to create an IT-like representation as a reweighted combination of the models. We fitted one weight for each of the 27 not-strongly-supervised models, the combi27 model, and the three SVM decision values. The weights were fitted by non-negative least squares, so as to minimize the sum of squared deviations between the RDM of the weighted combination of the features and the hIT RDM. The resulting weights are shown in the second row. Error bars indicate 95%-confidence intervals obtained by bootstrap resampling of the stimulus set. The resulting IT-geometry-supervised RDM is shown at the bottom (center) in juxtaposition to hIT (left) and mIT (right). Importantly, the RDM was obtained by cross-validation to avoid overfitting to the image set (Materials and Methods). The RDMs here are 92×92, excluding the four stimuli that we did not have monkey data for.

**Figure 6:**
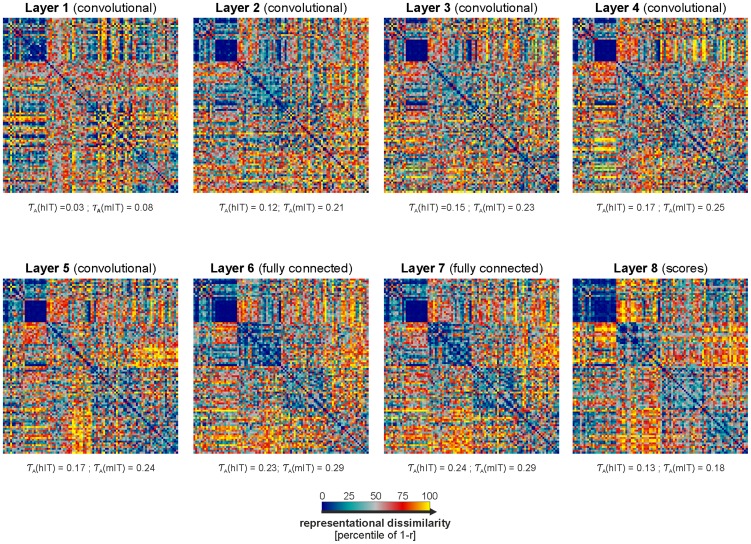
RDMs of all layers of the strongly supervised deep convolutional network. RDMs for all layers of the deep convolutional network (Krizhevsky et al. 2012) ref [41] are shown for the set of the 96 images (L1: layer 1 to L7: layer 7). Kendall-*τ*_A_ RDM correlations of the models with hIT and mIT are stated underneath each RDM. All correlations are statistically significant. For inferential comparisons to IT and other regions, see Figure 7 and Table 2, respectively.

**Figure 7:**
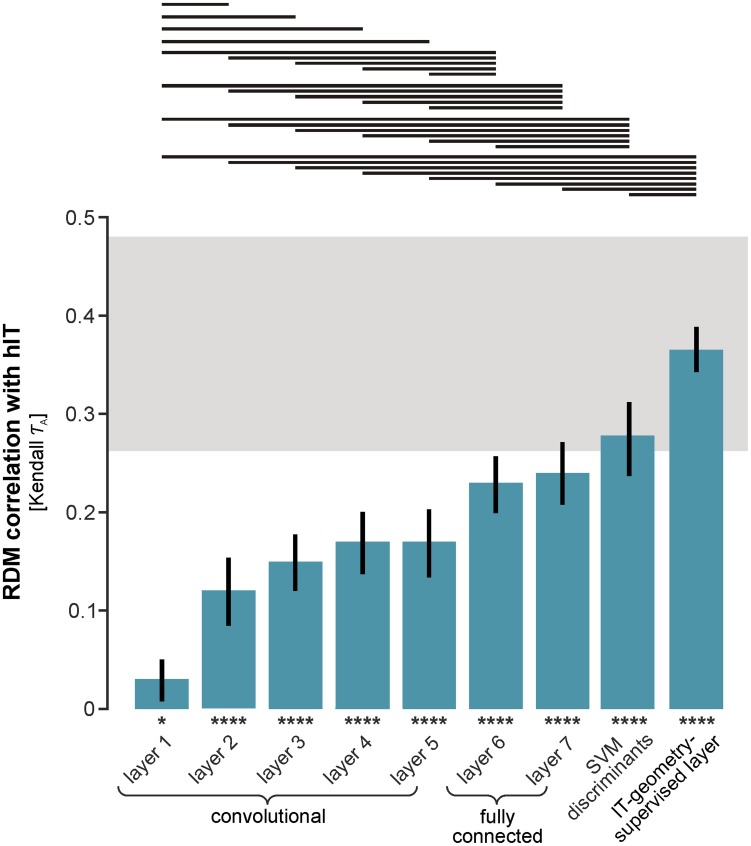
The strongly supervised deep network, with features remixed and reweighted, fully explains the IT data. The bars show the Kendall-τ_A_ RDM correlations between the layers of the strongly supervised deep convolutional network and human IT. The error bars are standard errors of the mean estimated by bootstrap resampling of the stimuli. Asterisks indicate significant RDM correlations (random permutation test based on 10,000 randomizations of the stimulus labels; p<0.05: *, p<0.01: **, p<0.001: ***, p<0.0001: ****). As we ascend the layers of the deep network, model RDMs explain increasing proportions of the variance of the hIT RDM. The noise ceiling (gray bar) indicates the expected correlation of the true model (given the noise in the data). The upper and lower edges of the gray horizontal bar are upper and lower bound estimates of the maximum correlation any model can achieve given the noise. None of the layers of the deep network reaches the noise ceiling. However, the final fully connected layers 6 and 7 come close to the ceiling. Remixing the features of layer 7 (Figure 10) using linear SVMs to strengthen the categorical divisions, provides a representation composed of three discriminants (animate/inanimate, face/nonface, and body/nonbody) that reaches the noise ceiling. Reweighting the model layers and the three discriminants (see Figure 10 for details) yields a representation that explains the hIT geometry even better. A horizontal line over two bars indicates that the two models perform significantly differently (inference by bootstrap resampling of the stimulus set). Multiple testing across the many pairwise comparisons is accounted for by controlling the expected FDR at 0.05. The pairwise statistical comparisons show that the IT-geometry-supervised deep model explains IT significantly better than all other candidate representations.

**Figure 8:**
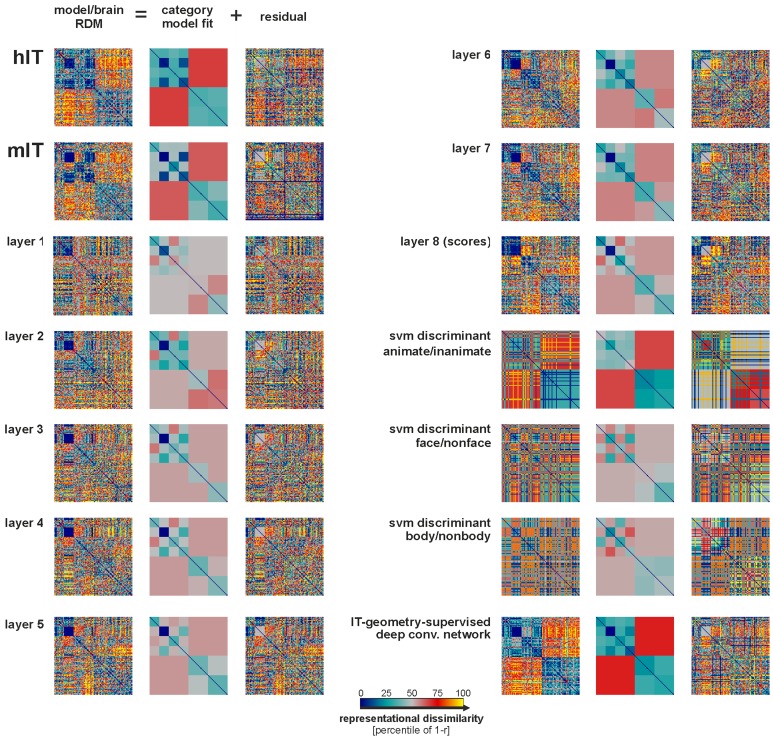
IT-like categorical structure emerges across the layers of the deep supervised model, culminating in the IT-geometry-supervised layer. Descriptive category-clustering analysis as in Figure 3, but for the deep supervised network. We used a linear combination of category-cluster RDMs (Figure S5) to model the categorical structure. The fitted linear-combination of category-cluster RDMs is shown in the middle columns. This descriptive visualization shows to what extent different categorical divisions are prominent in each layer of the deep supervised model. The layers show some of the categorical divisions emerging. However, remixing of the features (linear SVM readout) is required to emphasize the categorical divisions to a degree that is similar to IT. The final IT-geometry-supervised layer (weighted combination of layers and SVM discriminants) has a categorical structure that is very similar to IT. Overfitting to the image set was avoided by crossvalidation. For statistical inference, see Figure 9.

**Figure 9:**
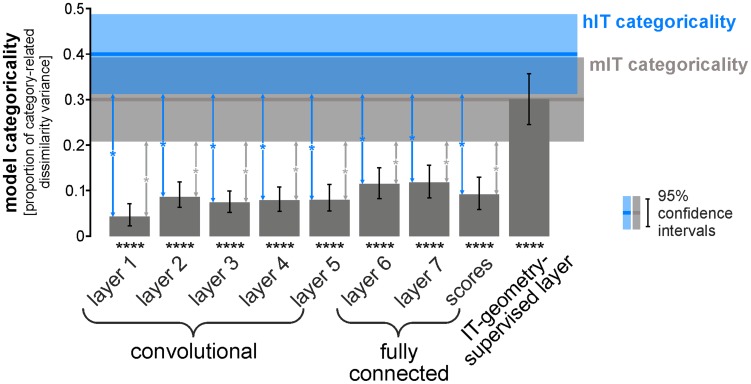
The layers of the deep supervised model are less categorical than IT, but remixing and reweighting achieves IT-level categoricality. Bars show the categoricality index for each layer of the deep convolutional network and for the IT-geometry-supervised layer. For conventions and for definition of the categoricality index, see Figure 4. Error bars and shaded regions indicate 95%-confidence intervals. Significant Categoricality indices are indicated by stars underneath the bars (* p<0.05, ** p<0.01, *** p<0.001, **** p<0.0001). Significant differences between the categoricality index of each model and the hIT categoricality index are indicated by blue vertical arrows (p<0.05, Bonferroni-adjusted for 9 tests). The corresponding inferential comparisons for mIT are indicated by gray vertical arrows. Categoricality is significantly greater in hIT and mIT than in any of the internal layers of the deep convolutional network. However, the IT-geometry-supervised layer (remixed and reweighted) achieves a categoricality similar to (and not significantly different from) IT. This analysis is based on equating the noise level in the models with that of hIT (Materials and Methods). Similar results obtain for a conservative inferential analysis comparing the categoricality of the noise-less models with that of the noisy estimates for hIT and mIT (Figure S10).

**Figure 10:**
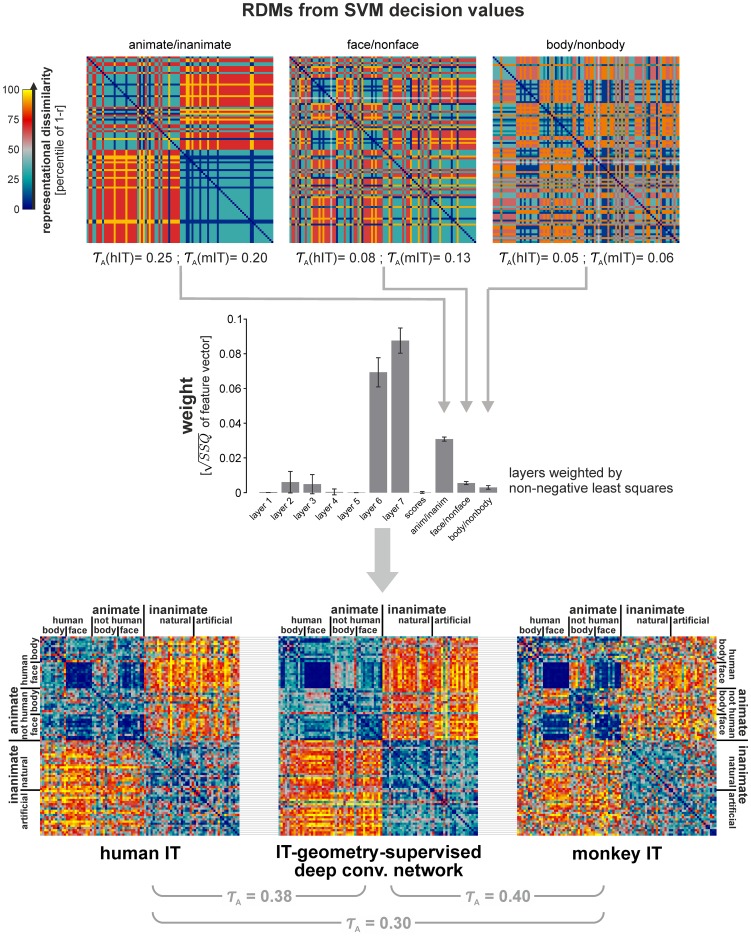
Remixing and reweighting features of the deep supervised network achieves an IT-like representational geometry. All analyses and conventions here are analogous to Figure 5, but applied to the strongly supervised deep convolutional network, rather than to the not-strongly supervised models. Remixing the features of layer 7 by fitting linear SVMs (separate set of training images) for the major categorical divisions (animate/inanimate, face/nonface, and body/nonbody) helped account for the categorical clusters in IT. The Kendall-*τ*_A_ RDM correlations between the SVM decision values and IT (stated underneath the RDMs in the top row) are statistically significant (p<0.05). For the deep convolutional network used here, feature remixing accounted for the animate/inanimate division of IT. We attempted to create an IT-like representation as a reweighted combination of the layers of the deep network and the SVM decision values. We fitted one weight for each of the layers and one weight for each of the three decision values. The bar graph in the middle row shows the weights, with 95%-confidence intervals obtained by bootstrap resampling of the stimulus set. As before, the weights were fitted using non-negative least squares to minimize the sum of squared deviations between the RDM of the weighted combination and the hIT RDM. The resulting IT-geometry-supervised RDM (bottom row, center) is very similar to the RDMs of hIT (left) and mIT (right). The *τ*_A_ RDM correlation between the fitted model and IT is about equal for monkey IT (0.40) and human IT (0.38). Both of these RDM correlations are higher than the RDM correlation between hIT and mIT, reflecting the effect of noise on the empirical RDM estimates. As in Figure 5, the fitted model RDM was obtained by cross-validation to avoid overfitting to the image set.

The results for the 37 model representations are presented separately for two sets of representations. The first set comprises the not-strongly-supervised representations (Figures 1–5). The second set comprises the layers of a strongly supervised deep convolutional network and an IT-like representation constructed by remixing and reweighting the features of the deep supervised model (Figures 6–10). The not-strongly-supervised set (Table 1) includes two supervised models: GMAX and Supervised HMAX (Materials and Methods). These were supervised much more weakly than the deep convolutional network, using merely hundreds of images. The deep convolutional network (Table 2) was supervised with 1.2 million category-labeled images. Note that the first set contains many independent model representations, whereas the second set contains the stages of a single deep strongly supervised object-vision model.

**Table 1:** RDM correlations between brain regions and not-strongly-supervised models.

			correlation to					
		model	mIT	hIT [0.26, 0.48]	LOC [0.20, 0.41]	FFA [0.10, 0.39]	PPA [0.08, 0.38]	EVC [0.13, 0.40]
**without training**		**1. V1 model**	0.104****	0.080***	0.048**	0.045*	0.006^ns^	0.123****
		**2.Convolutional network**	0.132****	0.111****	0.058***	0.083***	−0.023^ns^	0.174****
		**3.Bio transform-1^st^ stage**	0.117****	0.059**	0.031^ns^	0.023^ns^	0.018^ns^	0.075*
		**4.Bio transform-2^nd^ stage**	0.096****	0.015^ns^	0.029^ns^	0.077**	−0.008^ns^	0.044^ns^
		**5. Bio transform-both**	0.105****	0.056**	0.066***	0.067**	−0.026^ns^	0.037^ns^
		**6. Radon**	0.013^ns^	0.035^ns^	−0.002^ns^	−0.031^ns^	0.016^ns^	0.004^ns^
		**7. Dense SIFT**	0.145****	0.101****	0.077****	0.067***	0.021^ns^	0.171****
		**8. Stimulus image (Lab)**	0.044**	0.023^ns^	0.044*	0.083*	−0.033^ns^	−0.119^ns^
		**9. LBP**	0.067***	0.078**	0.084****	0.051*	−0.057^ns^	−0.025^ns^
		**10. Lab joint histogram**	0.147****	0.092****	0.081****	0.094***	−0.055^ns^	0.011^ns^
		**11. Silhouette image**	0.168****	0.092****	0.061***	0.052*	−0.003^ns^	0.209****
		**12. Gist**	0.164****	0.120****	0.065****	0.059**	−0.014^ns^	0.185****
		**13. PHOG**	0.120****	0.094****	0.059**	0.061*	−0.010^ns^	0.212****
		**14. Geometric blur**	0.123****	0.061***	0.028^ns^	0.058*	0.010^ns^	0.172****
		**15. Ssim**	0.171****	0.126****	0.068****	0.104***	−0.048^ns^	0.169****
		**16. Gssim**	0.158****	0.106****	0.069****	0.063**	−0.000^ns^	0.191****
**with training**	**un-supervised**	**17.VisNet_UT_**	0.012^ns^	0.037**	0.016^ns^	−0.005^ns^	−0.001^ns^	0.057***
		**18.Stable model_UT_**	0.092****	0.081****	0.056**	0.088**	−0.083^ns^	0.004^ns^
		**19.HMAX-C1_UT_**	0.127****	0.085****	0.051***	0.036^ns^	0.025^ns^	0.154****
		**20.HMAX-C2_UT_**	0.182****	0.114****	0.055***	0.098***	−0.012^ns^	0.217****
		**21.HMAX-C2b_UT_**	0.114****	0.112****	0.095****	0.065**	−0.043^ns^	−0.011^ns^
		**22.HMAX-C3_UT_**	0.139****	0.114****	0.074****	0.081***	−0.047^ns^	0.078**
		**23.HMAX-All_UT_**	0.165****	0.139****	0.108****	0.132****	−0.067^ns^	0.081**
		**24.SLF_UT_**	0.061****	0.054**	0.015^ns^	0.091***	−0.074^ns^	−0.027^ns^
		**25.PHOW_UT_**	0.031*	0.054*	0.046*	0.038^ns^	−0.067^ns^	−0.021^ns^
**with training**	**supervised**	**26.GMAX_ST_**	0.078****	0.060***	0.023^ns^	0.098***	−0.074^ns^	−0.038^ns^
		**27.Supervised HMAX_ST_**	0.085****	0.079****	0.041*	0.109***	−0.074^ns^	−0.032^ns^
		**28. Combination of all 27**	0.253****	0.169****	0.137****	0.045****	0.034***	0.096****

Kendall-*τ*_A_ RDM correlation coefficients between brain regions and not-strongly-supervised models. Significant correlations are indicated by asterisks (ns: not significant, * p<0.05, ** p<0.01, *** p<0.001, **** p<0.0001). The brain regions are the lateral occipital complex (LOC), the fusiform face area (FFA), the parahippocampal place area (PPA), and the foveal confluence of early visual areas (EVC). For each brain region, the highest RDM correlation is set in bold. Lower and upper bounds of the noise ceiling are stated in brackets below the labels to the human brain ROIs (top row).

**Table 2:** RDM correlations between brain regions and layers of the deep convolutional network.

		correlation to					
	model	mIT	hIT [0.26, 0.48]	LOC [0.20, 0.41]	FFA [0.10, 0.39]	PPA [0.08, 0.38]	EVC [0.13, 0.40]
**deep convolutional network layers** (Krizhevsky et al. 2012)	**Layer 1** (convolutional)	0.08*	0.03*	0.03**	0.04*	−0.03^ns^	0.01^ns^
	**Layer 2** (convolutional)	0.21****	0.12****	0.08****	0.09****	−0.01^ns^	0.17****
	**Layer 3** (convolutional)	0.23****	0.15****	0.10****	0.07***	−0.01^ns^	0.16****
	**Layer 4** (convolutional)	0.25****	0.17****	0.12****	0.06***	0.01^ns^	0.11****
	**Layer 5** (convolutional)	0.24****	0.17****	0.14****	0.05**	0.01^ns^	0.09****
	**Layer 6** (fully connected)	0.29****	0.23****	0.18****	0.12****	−0.02^ns^	0.07****
	**Layer 7** (fully connected)	0.29****	0.24****	0.18****	0.09****	−0.02^ns^	0.06**
	**Layer 8** (scores)	0.18****	0.13****	0.12****	0.02^ns^	−0.02^ns^	0.01^ns^
**remixed features** (linear SVM readout)	**animate/inanimate**	0.20****	0.25****	0.20****	0.02^ns^	0.07****	0.03*
	**face/nonface**	0.13****	0.08***	0.08***	0.04***	0.02**	0.03**
	**body/nonbody**	0.06**	0.05***	0.05***	0.00^ns^	0.01^ns^	−0.01^ns^
**reweighted combination of the above**	**IT-geometry supervised Layer**	0.40****	0.38****	0.27****	0.07****	0.05***	0.07****

Kendall-*τ*_A_ RDM correlation coefficients between brain regions and layers of the deep supervised network. Conventions as in Table 1. Significant correlations are indicated by asterisks (ns: not significant, * p<0.05, ** p<0.01, *** p<0.001, **** p<0.0001). For each brain region, the highest RDM correlation is set in bold.

### Most models explain a small component of the IT representational geometry

Among the not-strongly-supervised models, the seven models with the highest RDM correlations with hIT and mIT are shown in Figure 1 (for other brain regions, see Figure S1 and Table 1). Visual inspection suggests that the models capture the human-face cluster, which is also prevalent in IT. However, the models do not appear to place human and animal faces in a single cluster. In addition, the inanimate objects appear less clustered in the models.

All models shown in Figure 1 have small, but highly significant (p<0.0001) RDM correlations with hIT and mIT (Figure 1A, 1B, respectively; for RDM correlation with other brain regions see Figure S2 for the not-strongly-supervised models, and Figure S3 for the deep supervised model representations). Most of the other not-strongly-supervised models also have significant RDM correlations (Table 1, Figure 2; inference by randomization of stimulus labels). Although often significant, all RDM correlations between not-strongly-supervised models and IT were small (Kendall *τ*_A_<0.17 for hIT; *τ*_A_<0.26 for mIT).

### Combining features from multiple models improves the explanation of IT

Combining features from the not-strongly-supervised models improved the RDM correlations to IT. Model features were combined by summarizing each model representation by its first 95 principal components and then concatenating these sets of principal components. This approach ensured that each model contributed equally to the combination (same number of features and same total variance contributed).

The combination of the 27 not-strongly-supervised models (combi27) has a higher RDM correlation with both hIT and mIT than any of the 27 contributing models. Second to the combi27 model, internal representations of the HMAX model have the highest RDM correlation with hIT and mIT. This might reflect the fact that the architecture and parameters of the HMAX model closely follow the literature on the primate ventral stream.

In addition to the combi27, we also tested the combination of untrained models, the combination of unsupervised trained models, and the combination of weakly supervised trained models (Figure S4). The combi27 explained IT equally well or better than other combinations of the not-strongly-supervised models. In the remaining analyses, we therefore omit the other combinations and consider the combi27 along with each individual model.

Monkey IT was significantly better explained by the combi27 than by the second best among the not-strongly-supervised models (HMAX-C2_UT_; p = 0.02; inference by bootstrap resampling of the stimulus set [50], not shown). This suggests that the models are somewhat complementary in explaining the IT features space. For hIT, the second best model was also a version of HMAX (HMAX-all_UT_), but it did not explain hIT significantly worse than combi27 (p = 0.261, not shown).

Model RDM correlations with mIT tended to be higher than model correlations with the hIT RDM. For example, the dissimilarity correlation of the combi27 with mIT was 0.25, whereas for hIT it is 0.17. This difference is statistically significant (p = 0.001), suggesting that the models were able to better explain the mIT RDM compared to the hIT RDM. This could be caused by a lower level of noise in the mIT RDM (estimated from cell-recording data) than in the hIT RDM (from fMRI data).

### None of the not-strongly-supervised models fully explains the IT data

For the human data we were able to estimate a noise ceiling [30] (Materials and Methods), indicating the RDM correlation expected for the true model, given the noise in the data. None of the 28 not-strongly-supervised models reached the noise ceiling (Figure 2A). The combi27 representation came closest, but at *τ*_A_ = 0.17, it was far from the lower bound of the noise ceiling (*τ*_A_ = 0.26). This indicates that the fMRI data capture a component of the hIT representation that all the not-strongly-supervised models leave unexplained. For mIT, we could not estimate the noise ceiling because we had data from only two animals.

### IT is more categorical than any of the not-strongly-supervised models

The main categorical divisions observed in IT appear weak or absent in the best fitting models (Figure 1). To measure the strength of categorical clustering in each model and brain representation, we fitted a linear model of category-cluster RDMs to each model and brain RDM (Materials and Methods, Figure S5). The fitted models (Figure 3) descriptively visualize the categorical component of each RDM, summarizing sets of within- and between-category dissimilarities by their averages. The fits for several computational models show a strong human-face cluster, and a weak animate cluster. The human-face cluster is expected on the basis of the visual similarity of the human-face images (all frontal aligned human faces of the same approximate size). The animate cluster could reflect the similar colors and more rounded shapes shared by the animate objects. However, IT in both human and monkey exhibits additional categorical clusters that are not easily accounted for in terms of visual similarity. First, the IT representation has a strong face cluster that includes human and animal faces of different species, which differ widely in shape, color, and pose. Second, the IT representation has an inanimate cluster, which includes a wide variety of natural and artificial objects and scenes of totally different visual appearance. These clusters are largely absent from the not-strongly-supervised models (Figures 3, S6, S7, S8).

In order to statistically compare the overall strength of categorical divisions between IT and each of the models, we computed a categoricality index for each representation. The categoricality index is the proportion of RDM variance explained by categorical divisions. The categoricality index is calculated as the squared correlation between the fitted category-cluster model (Figure S5) and the RDM it is fitted to (Figure 4). The model RDMs are noise-less. However, the brain RDMs are affected by noise, which lowers the categoricality index. To account for the noise and make the categoricality indices comparable between models and IT, we added noise matching the noise level of hIT to the model representations (Materials and Methods). We then compared the categoricality indices of the 28 not-strongly-supervised models to that of hIT (Figure 4). Human IT has a categoricality index of 0.4. All of the not-strongly supervised models have categoricality indices below 0.16; most of them below 0.1.

Inferential comparisons show that the categoricality index is significantly higher for hIT than for any of the models (inference by bootstrap resampling of the image set). We also compared the categoricality indices between models and IT without equating the noise levels. In this analysis, the categoricality index reflects the categoricality of the models without noise. For hIT and mIT, the noise lowers the categoricality estimate. Nevertheless, the hIT categoricality index remains significantly greater than that of any of the models. For mIT, similarly, the categoricality index is significantly greater than for all but three of the models (Figure S9).

We also analyzed the clustering strength separately for each of the categories (Figure S6). For animates, clustering strength was significant for a few models (Lab joint color histogram, PHOG, and HMAX-all). For human faces, significant clustering was observed for several computational models (convNet, bioTransform, dense SIFT, LBP, silhouette image, gist, geometric blur, local self-similarity descriptor, global self-similarity descriptor, stable model, HMAX-C1, and combi27). These significant category clusters reflect the visual similarity of the members of these categories.

Inferential comparisons of clustering strength between each of the models and hIT (Figure S8) and mIT (Figure S8) for each of the categories revealed that IT clusters animates, inanimates, and faces (including human and animal faces) significantly more strongly in both species than most of the models (blue bars in Figures S7 and S8). There are only a few cases, in which a model clusters one of the categories more strongly than IT.

### Remixing and reweighting of the features of the not-strongly-supervised models does not improve the explanation of the IT data

The finding that categoricality is stronger in IT than in any of the models raises the question of what the models are missing. One possibility is that the models contain all essential nonlinear features, but in proportions different from IT, thus emphasizing the features differently in the representational geometry. In that case reweighting of the features (i.e. stretching and squeezing the representational space along its original axes) should help approximate the IT representational geometry.

For example, the representation might contain a feature perfectly discriminating animates from inanimates. This single categorical feature would not have been reflected strongly in the overall RDM if none of the other features emphasized this categorical division. The influence of such a feature on the overall representational geometry could be increased either by replicating the feature in the representation or by amplifying the feature values. These two alternatives are equivalent in their effects on the RDM, so we consider only the latter.

Another possibility is that all essential nonlinearities are present, but the features need to be linearly recombined (i.e. performing general affine transformations) to approximate the IT representational geometry. We therefore investigated whether linear remixing and reweighting of the features of the not-strongly-supervised models could provide a better explanation of the IT representational geometry.

#### Remixing of features

**Figure 11:**
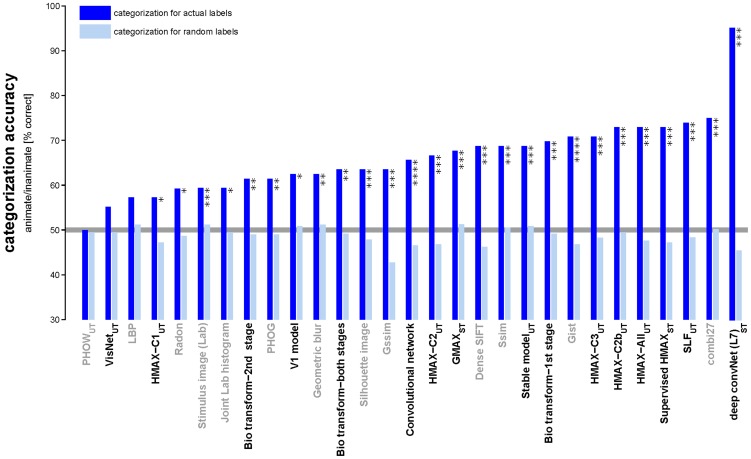
Animate/inanimate categorization accuracy for all models. Each dark blue bar shows the categorization accuracy of a linear SVM applied to one of the computational model representations. Categorization accuracy for each model was estimated by 12-fold crossvalidation on the 96 stimuli. To assess whether categorization accuracy was above chance level, we performed a permutation test, in which we retrained the SVMs on (category-orthogonalized) 10,000 random dichotomies among the stimuli. Light blue bars show the average model categorization accuracy for random label permutations. Categorization performance was significantly greater than chance for most models (* p<0.05, ** p<0.01, *** p<0.001, **** p<0.0001). The deep convolutional network model (final fully connected layer 7) has the highest animate/inanimate categorization performance (96%). The combi27 has the second highest performance (76%).

We attempted to create new features as linear combinations of the original features. The space of all linear recodings is difficult to search given limited data. We therefore restricted this analysis to the combi27 features (which represent a combination of the not-strongly-supervised models) and attempted to find linear combinations that specifically emphasize the missing categorical divisions. In order to find such linear combinations, we trained three linear support vector machine (SVM) classifiers for body/nonbody, face/non-face, and animate/inanimate categorization. The SVMs were trained on a set of 884 labeled images of isolated objects nonoverlapping with the set of 96 images we had brain data for. We used the decision-value outputs of the classifiers as new features. The resulting single-feature RDMs (Figure 5, top; one RDM for each SVM) are not highly categorical and have only a low correlation (*τ*_A_<0.1) with the IT RDMs for human and monkey. This is consistent with the fact that the combi27 representation does not perform very well on categorization tasks (Figures 11, S11).

#### Feature reweighting

Combining the not-strongly-supervised models with equal weight in the combi27 representation improved the explanation of our IT data. We wanted to test whether appropriate weighting of the not-strongly-supervised models could further improve the explanation of the IT geometry. In addition to the 27 not-strongly-supervised models, we included the combi27 model, and the three categorical SVM discriminants in the set of representations to be combined. We fitted one weight for each of these representations (27+1+3 = 31 weights in total), so as to best explain the hIT RDM (Figure 5, middle row).

Flipping the sign of a feature (weight = −1) has no effect on the representational distances. We can, thus, consider only positive weights, without loss of generality. We therefore used a non-negative-least-squares fitting algorithm [51] to find the non-negative weights for the models that minimize the sum of squared deviations between the hIT RDM and the RDM of the weighted combination of models. The RDM of the weighted combination of the model features is equivalent to a weighted combination of the RDMs of the models (Materials and Methods) when squared Euclidean distance is used. We used the squared Euclidean distance for normalized representational patterns, which is equivalent to correlation distance, as used throughout this paper. We therefore applied the nonnegative least-squares algorithm at the level of the RDMs.

In order to avoid overestimation of the RDM correlation between the fitted model and hIT due to overfitting to the image set, we fitted the weights to random subsets of 88 of the 96 images in a crossvalidation procedure, holding out 8 images on each fold. We then estimated the representational dissimilarities for the weighted-combination model for the 8 held-out images. We repeated this procedure until the entire RDM of 96 by 96 images was estimated (Figure 5, bottom row, center).

Feature reweighting and remixing did not reproduce the categorical structure observed in IT (Figure 5, bottom row). In fact the weighted-combination model did slightly worse than combi27 at explaining hIT and mIT (*τ*_A_ = 0.13 for hIT, *τ*_A_ = 0.20 for mIT). The lower performance, despite the inclusion of combi27 as one of the component representations, reflects the cost of overfitting. However, since we fitted only 31 weights in the reweighting step, that cost is small. The failure to improve the explanation of the IT geometry through remixing and reweighting, thus, suggests that the not-strongly-supervised models are missing features important to the IT representational geometry. Different nonlinear features and more powerful supervised learning methods may be needed to fully capture the structure of the IT representation. We therefore next tested a deep supervised convolutional neural network [52].

### A strongly supervised deep convolutional network better explains the IT data

So far, we showed that none of the not-strongly-supervised models were able to reproduce the categorical structure present in IT. Most of these models were untrained or trained without supervision. A few of them were weakly supervised (i.e. supervised with merely 884 training images). Their failure at explaining our IT data suggests that computational features trained to cluster the categories through supervised learning with many labeled images might be needed to explain the IT representational geometry. We therefore tested a deep convolutional neural network trained with 1.2 million labelled images [52], nonoverlapping with the set of 96 images used here. The model has eight layers. The RDM for each of the layers and the RDM correlations with hIT and mIT are shown in Figure 6. The deep supervised convolutional network explains the IT geometry better than any of the not-strongly-supervised models. The RDM correlation between hIT and the deep convolutional network's best-performing layer (layer 7) is *τ*_A_ = 0.24. Layer 7 explains the hIT representation significantly better (p<0.05; obtained by bootstrap resampling of the stimulus set) than combi27 (*τ*_A_ = 0.17), the best-performing of the not-strongly-supervised models. Monkey IT, as well, is better explained by layer 7 (*τ*_A_ = 0.29) than by combi27 (*τ*_A_ = 0.25), although the difference is not significant.

Layer 7 is the deep network's highest continuous representational space, followed only by the readout layer (layer 8, also known as the “scores”). The readout layer is composed of 1000 features, one for each of the 1000 category labels used in training the network. The readout layer has a lower RDM correlation with hIT (*τ*_A_ = 0.13) and mIT (*τ*_A_ = 0.18) than layer 7.

From layer 1 to layer 7 the RDM correlation with IT rises roughly monotonically (Figure 7, Table 2) and many of the pairwise comparisons between RDM correlations for higher and lower layers are significant (Figure 7, horizontal lines at the top). Nevertheless, even the best-performing layer 7 does not reach the noise ceiling (Figure 7). Although the deep convolutional network outperforms all not-strongly-supervised models, it does not fully explain our IT data.

As for the not-strongly-supervised models, we analyzed the categoricality of the layers of the deep supervised model (Figures 8, 9). All layers of the deep supervised model, including layer 7 and layer 8 (the readout layer), have significantly lower categoricality indices than hIT and mIT (Figure 9). This might reflect the fact that the stimulus set was equally divided into animates and inanimates and this division, thus, strongly influences our categoricality index. Importantly, the deep supervised network emphasizes some categorical divisions more strongly and others less strongly than IT (Figure 8). For example, layer 7 emphasizes the division between human and animal faces and the division between artificial and natural inanimate objects more strongly than IT. However, IT emphasizes the animate/inanimate and the face/body division more strongly than layer 7.

### Remixing and reweighting of the deep supervised features fully explains the IT data

We have seen that the deep supervised model provides better separation of the categories than the not-strongly-supervised models and that it also better explains IT. However, it did not reach the noise ceiling. As for the not-strongly-supervised models, we therefore asked whether remixing the features linearly (by adding linear readout features emphasizing the right categorical divisions) and reweighting of the different layers and readout features could provide a better model of the IT representation.

The method for remixing and reweighting was exactly the same as for the not-strongly-supervised models (Figure 5). However, the linear SVM features were based on layer 7 (instead of combi27) and the reweighting involved fitting one weight for each of the layers (1–8) and one weight for each of the three linear SVM features.

As before, the linear SVM features were trained for body/nonbody, face/non-face, and animate/inanimate categorization using the nonoverlapping set of 884 training images. The RDMs for the SVM readout features show strong categorical divisions (Figure 10, top row). This is consistent with the fact that the layer-7 representation performs well on categorization tasks (Figures 11, S11).

As before, we used non-negative least square fitting to find the weighted combination of the representations that best approximates hIT. Again, we avoided overfitting to the image set by fitting the weights to random subsets of 88 of the 96 images in a crossvalidation procedure, holding out 8 images on each fold. This procedure yielded a weight for each of the eight layers of the deep network and for each of the three linear SVM readout features (11 weights in total; Figure 10, middle row; Materials and Methods).

We refer to this weighted combination as the IT-geometry-supervised deep model. Inspecting the RDM reveals the similarity of its representational geometry to hIT and mIT (Figure 10, bottom row). The model emphasizes the major categorical divisions similarly to IT (Figure 8, bottom right). In contrast to all other models, this model has a categoricality index matching mIT and not significantly different from either mIT or hIT (Figure 9). The IT-geometry-supervised deep model explains hIT better than any layer of the deep network (Figure 7, horizontal lines at the top). It has the highest RDM correlation with hIT (*τ*_A_ = 0.38) and mIT (*τ*_A_ = 0.4) among all model representations considered in this paper. Importantly, it falls well within the upper and lower bounds of the noise ceiling and, thus, fully explains the non-noise component of our hIT data.

### Model representations more similar to IT categorize better

Figure 11 shows the animate/inanimate categorization accuracy of linear SVM classifiers taking each of the model representations as their input (for the face/body dichotomy and the artificial/natural dichotomy among inanimates, see Figure S11). The categorization accuracy for each model was estimated by 12-fold crossvalidation of the 96 stimuli (Materials and Methods). The deep convolutional network model (layer 7) has the highest animate/inanimate categorization performance (96%), and the combi27 has the second highest performance (76%).

**Figure 12:**
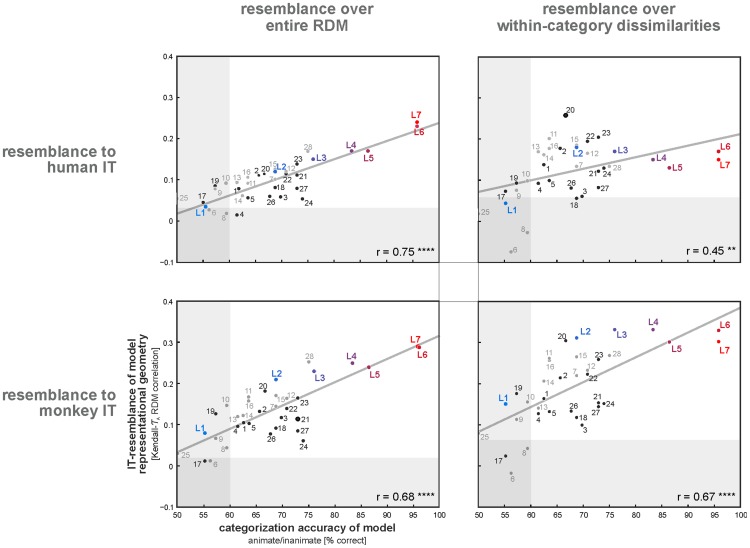
Model representations resembling IT afford better categorization accuracy. A model's IT-resemblance (measured by the RDM correlation between IT and model) predicts its categorization accuracy (animate/inanimate). This holds for both human-IT resemblance (top) and monkey-IT resemblance (bottom). The substantial positive correlation between IT-resemblance and categorization accuracy could reflect the categorical clustering of IT (left panels). However, the within-category RDM correlation between a model and IT also predicts model categorization accuracy (right panels). Each panel shows the least-squares fit (gray line) and the Spearman rank correlation r (* p<0.05, ** p<0.01, *** p<0.001, **** p<0.0001). Each circle shows one of the models. Numbers indicate the model (see Table 1 for model numbering). Different layers of the deep supervised convolutional network are indicated by colored labels “L1” (layer 1) to “L7” (layer 7). The deep model's layers are color-coded from light blue to light red (from lower to higher layers). Computer vision models are shown by gray circles; biologically motivated models are shown by black circles. The transparent horizontal and vertical rectangles cover non-significant ranges along each axis.

Figure 12 shows that models whose representations were more similar to IT tended to have a higher animate/inanimate categorization performance. The Pearson correlation between the IT-to-model representational similarity (*τ*_A_ RDM correlation) and categorization accuracy was 0.75 for hIT and 0.68 for mIT across the 28 not-strongly-supervised model representations and the seven layers of the deep supervised model. This finding could simply reflect the fact that the categories correspond to clusters in the IT representation and any representation clustering the categories will be well-suited for categorization. Indeed categorization performance is also predicted by the RDM correlation between a model and an animate-inanimate categorical RDM, albeit with a lower correlation coefficient (r = 0.38, not shown).

In order to further assess whether it was only the category clustering that predicted categorization accuracy or something deeper about the similarity of the model representation to IT, we considered the within-category dissimilarity correlation between each model and IT as a predictor of categorization accuracy. Models that were more similar to IT in terms of their within-category representational geometry (dissimilarities among animates and dissimilarities among inanimates) also tended to have higher categorization performance (Pearson r = 0.45 for hIT, r = 0.67 for mIT; p<0.01, p<0.0001, respectively).

These results may add to the motivation for computer vision to learn from biological vision. If computational feature spaces more similar to the IT representation yield better categorization performance within the present set of models, then it might be a good strategy for computer vision to seek to construct features even more similar to IT.

### Several models using Gabor filters and other low-level features explain human early visual cortex

We could not distinguish early visual areas V1, V2, and V3, because stimuli were presented foveally in the human fMRI experiment (2.9° visual angle in diameter, centered on fixation). Instead we defined an ROI for early visual cortex (EVC), which covered the foveal confluence of these retinotopic representations.

Several models using Gabor filters (SIFT, gist, PHOG, HMAX, ConvNet) and other features (Geometric blur, local self-similarity descriptor, global self-similarity descriptor, silhouette image) explained the early visual RDM estimated from fMRI (Figure S1A, S2A). These models not only explained significant dissimilarity variance, but reached the noise ceiling, indicating that they explain the EVC representation to the extent that the noise in our data enables us to assess this. For the HMAX model (as implemented by Serre et al. [20]), we tested several internal representations. The HMAX-C2 layer had the highest RDM correlation with EVC among all models. The HMAX-C2 layer falls within the early stages (above S1, C1, and S2 layers, and below S2b, S3, C2b, C3, and S4 layers) of the HMAX model and its features closely parallel the initial stages of primate visual processing. For the deep supervised model, the RDM correlations of different layers with EVC are shown in Figure S3A. Layers 2 and 3 of the model have the highest RDM correlation with EVC and reach the noise ceiling. However, their correlation with EVC is lower than that of the HMAX-C2 layer.

### Object-vision models and other brain regions

We also compared the model RDMs with brain areas other than IT and EVC (i.e. FFA, LOC, and PPA). Figure S2 shows how well each of the 28 not-strongly-supervised models explained EVC, FFA, LOC, and PPA. The seven not-strongly-supervised models with the highest RDM correlations to these brain regions are shown in Figure S1. Among the not-strongly-supervised models, the HMAX model showed the highest RDM correlation with EVC and FFA. Specifically, the HMAX-C2 layer had the highest RDM correlation with EVC (*τ*_A_ = 0.22) and HMAX-all had the highest RDM correlation with the FFA (*τ*_A_ = 0.13). The combi27 model had the highest RDM correlation with LOC and PPA (*τ*_A_ = 0.14 and *τ*_A_ = 0.03, respectively).

For the deep supervised model, Figure S3 shows how well different layers explain EVC, FFA, LOC, and PPA. Layers 2 and 3 reached the noise ceiling for EVC. Subsequent layers along the deep network's processing stream exhibited decreasing RDM correlations with EVC and increasing RDM correlations with LOC. Layer 7 gets closest to the LOC noise ceiling, but does not reach it. For FFA, however, layer 6 reaches the noise ceiling.

PPA exhibited the lowest RDM correlations with the models, including both the not-strongly-supervised and the deep supervised representations. The only model with a significant RDM correlation with PPA was combi27 (*τ*_A_ = 0.034, p<0.001; Table 1), which was far below the noise ceiling. This somewhat puzzling result might reflect a limitation of our stimulus set for investigating PPA. Konkle and Oliva [53] have shown that a bilateral parahippocampal region that overlaps with PPA responds more strongly to objects that are big than to objects that are small in the real world. Our stimulus set included a limited set of place and scene images and mostly objects that are small in the real world.

## Materials and Methods

### Object-vision models

We used a wide range of computational models to explore many different ways for extracting visual features. We selected some of the well-known biologically motivated object recognition models as well as several models and feature extractors from computer vision. Some of the models need a training phase (these are shown by a subscript –either ‘ST’ for supervised trained, or ‘UT’ for unsupervised trained) and some others do not (models without any subscript).

For the models with a training phase, we used a new set of 884 training images. Half of the images were animates and the other half were inanimates. Then, all models were tested using the testing stimuli (the set of 96 images). In the training set –similar to the testing set– animate images had subcategories of human/animal faces and human/animal bodies. Inanimate images had subcategories of artificial and natural inanimates.

Below is a description for all models used in this study (see [24] for a more comprehensive explanation of the models). For those models that the code was freely available online, we have provided the link.

#### Stimulus image (Lab)

Lab color space approximates a linear representation of human perceptual color space. Each Lab image was obtained by transferring the color image (175×175) from RGB color space to the Lab color space. Then, the image was converted to a pixel vector with the length of 175×175×3.

#### Color set (Lab joint histogram)

First, images (175×175) were transferred from RGB color space to Lab color space. Then, the three Lab dimensions were divided into 6 bins of equal width. The joint histogram was computed by counting the number of figure pixels falling into each of the 6×6×6 bins. Finally, the obtained lab joint histogram was converted to a vector with the length of 6×6×6.

#### Radon

The Radon transform of an image is a matrix, in which each column corresponds to a set of integrals of the image intensities along parallel lines of a given angle. The Matlab function Radon was used to compute the Radon transform for each luminance image.

#### Silhouette image

All RGB color images were converted to binary silhouette images by setting all background pixels to 0 and all figure pixels to 1. Each image was then converted to a vector with the length of 175×175.

#### Unsupervised convolutional network

A hierarchical architecture of two stages of feature extraction, each of which is formed by random convolutional filters and subsampling layers [39]. Convolutional layers scan the input image inside their receptive field. Receptive Fields (RFs) of convolutional layers get their input from various places on the input image, and RFs with identical weights make a unit. The outputs of each unit make a feature map. Convolutional layers are then followed by subsampling layers that perform a local averaging and subsampling, which make the feature maps invariant to small shifts [40]. The convolutional network which we used had two stages of unsupervised random filters, that is shown by RR in table 1 in Jarret et al. (2009) [39]. The obtained result for each image was then vectorized. The parameters were exactly the same as used in [39] (http://koray.kavukcuoglu.org/code.html).

#### Deep supervised convolutional network

This is a supervised convolutional neural network, trained with 1.2 million labelled images from ImageNet (1000 category labels) [52]. The network has 8 layers: 5 convolutional layers, followed by 3 fully connected layers. The output of the last layer is a distribution over the 1000 class labels. This is the result of applying a 1000-way softmax on the output of the last fully connected layer [54] [http://caffe.berkeleyvision.org/ (Caffe: Convolutional Architecture for Fast Feature Embedding)].

#### Biological Transform (BT)

BT is a hierarchical transform based on local spatial frequency analysis of oriented segments. This transform has two stages, each of which has an edge detector followed by an interval detector [38]. The edge detector consists of a bar edge filter and a box filter. For a given interval *I* and angle *θ*, the interval detector finds edges that have angle *θ* and are separated by an interval *I*. In the first stage, for any given *θ* and *I*, all pixels of the filtered image were summed and then normalized by the squared sum of the input. They were then rectified by the Heaviside function. The second stage was the same as the first stage, except that in the first stage *θ* was changing between 0–180° and *I* between 100–700 pixels and the input to the first stage had not a periodic boundary condition on the *θ* axis (repeating the right-hand side of the image to the left of the image and vice versa); but in the second stage the input, which is the output of the first stage, was given a periodic boundary condition on the *θ* axis, and *I* was changing between 15–85 pixels.

#### Gist

Each image was divided into 16 bins, and then oriented Gabor filters (in 8 orientations) were applied over different scales (4 scales) in each bin. Finally, the average filter energy in each bin was calculated [42], [55]. Then each obtained image was converted to a vector of length (8×8×8). The code is available from here: http://people.csail.mit.edu/torralba/code/spatialenvelope/

#### Geometric Blur (GB)

289 uniformly distributed points were selected on each image, then the Geometric Blur descriptors [56]–[58] were calculated by applying spatially varying blur around the feature points. We used GB features that were part of multiple kernels for image classification described in [59](http://www.robots.ox.ac.uk/~vgg/software/MKL/#download). The blur parameters were set to α = 0.5 and β = 1; the number of descriptors was set to 300.

#### Dense SIFT

For each grayscale image, SIFT descriptors [60] of 16×16 pixel patches were sampled uniformly on a regular grid. Then, all the descriptors were concatenated in a vector as the SIFT representation of that image. We used the dense SIFT descriptors that were used in [44] to extract PHOW features, described below.

#### Pyramid Histogram of Visual Words (PHOW_UT_)

Dense SIFT descriptors were calculated for each image and then quantized using k-means clustering to form a visual vocabulary. A spatial pyramid of three levels was then created and the histogram of SIFT visual words was calculated for each bin. The concatenation of all histograms was used as the PHOW representation of that image [44]. We used the implementation available online(http://www.cs.unc.edu/~lazebnik/research/spatial_pyramid_code.zip). The dictionary size was fixed to 200 and the number of spatial pyramid levels was fixed to three.

#### Pyramid Histogram of Gradients (PHOG)

The canny edge detector was applied on grayscale images, and then a spatial pyramid was created with four levels [45]. The histogram of orientation gradients was calculated for all bins in each level. All histograms were then concatenated to create PHOG representation of the input image. We used Matlab implementation that was freely available online (http://www.robots.ox.ac.uk/~vgg/research/caltech/phog.html). Number of quantization bins was set to forty, number of pyramid levels to four and the angular range to 360°.

#### VisNet _UT_

VisNet is a hierarchical model of ventral visual pathway for invariant object recognition that has four successive layers of self-organizing maps. Neurons which are higher in the hierarchy have larger receptive fields. Each layer in the model corresponds to a specific area of the primate ventral visual pathway in terms of the size of its receptive fields [34], [61]. The model was trained with trace learning rule [62]. The learning rate was set to 0.1 and number of epochs in each of the four layers was fixed to 100. Finally the representation of the last layer was vectorized and used as VisNet features.

#### Local self-similarity descriptor (ssim)

This is a descriptor that is not directly based on the image appearance; instead, it is based on the correlation surface of local self-similarities. For computing local self-similarity features at a specific point on the image, say *p*, a local internal correlation surface can be created around *p* by correlating the image patch centred at *p* to its immediate neighbours [46], [63]. We used the code available for ssim features that were part of multiple kernels for image classification described in [59](http://www.robots.ox.ac.uk/~vgg/software/SelfSimilarity/). The ssim descriptors were computed uniformly at every five pixels in both X and Y directions.

#### Global self-similarity descriptor (gssim)

This descriptor is an extension of the local self-similarity descriptor mentioned above. Gssim uses self-similarity globally to capture the spatial arrangements of self-similarity and long range similarities within the entire image [47]. We used gssim Matlab implementation available online(http://www.vision.ee.ethz.ch/~calvin/software.html). Number of clusters for the patch prototype codebook was set to 400, with 20000 patches to be clustered. D1 and D2 for the self-similarity hypercube were both set to 10.

#### Local Binary Patterns (LBP)

Local binary patterns are usually used in texture categorization. The underlying idea of LBP is that a 2-dimensional surface can be described by two complementary measures: local spatial patterns and gray scale contrast. For a given pixel, LBP descriptor gives binary labels to surrounding pixels by thresholding the difference between the intensity value of the pixel in the center and the surrounding pixels [48], [64], [65]. We used LBP Matlab implementation freely available online(http://www.cse.oulu.fi/CMV/Downloads/LBPMatlab). Number of sampling points was fixed to eight.

#### V1 model

A population of simple and complex cells were modelled and were fed by the luminance images as inputs. Gabor filters of 4 different orientations (0°, 90°, −45°, and 45°) and 12 sizes (7–29 pixels) were used as simple cell receptive fields. Then, the receptive field of complex cells were modelled by performing the MAX operation on the neighboring simple cells with similar orientations. The outputs of all simple and complex cells were concatenated in a vector as the V1 representational pattern of each image.

#### HMAX_UT_

The HMAX model developed by Serre et al. [20] has a hierarchical architecture inspired by the well-known simple to complex cells model of Huble & Wiesel [66], [67]. There has been several extensions to the HMAX model, improving its feature selection process (e.g. [37]) or adding new processing layers to the model [68]. The HMAX model that is used here adds three more layers –ends at S4- on the top of the complex cell outputs of the V1 model described above. The model has alternating S and C layers. S layers perform a Gaussian-like operation on their inputs, and C layers perform a max-like operation, which makes the output invariant to small shifts in scale and position. We used the freely available version of the HMAX model (http://cbcl.mit.edu/software-datasets/pnas07/index.html). All simple and complex layers were included until the S4 layer.

Note: The HMAX model which has been used in [18] was a pre-trained version of the HMAX model; however, in this study we have trained the HMAX model using a dataset that contains 442 animate and 442 inanimate objects. So, the obtained RDMs are different.

#### Sparse Localized Features (SLF_UT_)

This is a biologically motivated model based on the HMAX C2 features. The model introduces sparsified and localized intermediate-level visual features [33]. We used the Matlab code available for these feature (http://www.mit.edu/~jmutch/fhlib/); and the default model parameters were used.

#### GMAX_ST_

This model is an extension of the HMAX model C2 features in which authors have used feedback from the classification layer (analogous to PFC) to extract informative visual features. Their method uses an optimization algorithm (i.e. genetic algorithm) to select informative patches from a large pool of patches [35]. Using genetic algorithm a subset of patches that gives the best categorization performance is selected. A linear SVM classifier was used to calculate the categorization performance. In other words, in the training phase of the model the categorization performance is used as the fitness function for the genetic algorithm. To run this model we used the same set of model parameters suggested in [35]. In the process of finding optimal patches in the optimization algorithm, we used a random subset of 884 training images described before.

#### Stable Model _UT_

This is another biologically motivated model, which has a hierarchy of simple to complex cells. The model uses the adaptive resonance theory (ART) mechanism [69] for extracting informative intermediate level visual features. This has made the model stable against forgetting previously learned patterns [36]. Similar to HMAX model it extracts C2-like features, except that in the training phase it only selects the highest active C2 units as prototypes that represent the input image. This is done using top-down connections from C2 layer to C1 layer. The connections match the C1-like features of the input image to the prototypes of the C2 layer. The matching degree is controlled by a vigilance parameter that is fixed separately on a validation set. We set the model parameters the same as was suggested by authors except that instead of using all patch sizes, we used patches of size 12 that made the output RDM more correlated with brain RDMs. It is also shown in [36] that patches of size 12 make the model more stable. Furthermore when using patches of size 12, the model performs better in the face/non-face classification task [36].

#### Supervised HMAX_ST_

We used this approach to remove non-discriminative patches of the HMAX model. After training the HMAX model with the training images of animates and inanimates, extracted patches were divided into two clusters using k-means clustering. One cluster represented the patches extracted from animate images, and the other cluster represented the patches extracted from inanimate images. Then, in order to remove the non-discriminative patches (i.e. patches that do not distinguish between animates and inanimates), those patches that were extracted from the animate images but fell nearer to the center of the inanimate cluster were removed. Similarly the patches that were extracted from the inanimate images but fell nearer to the center of the animate cluster were removed. The remaining patches were used for the test phase.

#### Combination of all not-strongly-supervised models (combi27)

This is the concatenation of features extracted by all of the above-mentioned models. Given an input stimulus, features from all of the above-mentioned models were extracted. Because the dimension for extracted features differs across models, we used principle component analysis (PCA) to reduce the dimension of all of them to a unique number. We used the first 95 PCs from each of the models and concatenated them along a vector (95 was the largest possible number of PCs that we were able to use, because we had 96 images; so the covariance matrix has only 95 non-zero eigenvalues). Therefore, combi27 features for each image is a vector of length 95×27 = 2565.

For some of the above-mentioned models that had a hierarchical architecture, we made an RDM for each of the stages in the hierarchy, as well as an RDM from the concatenation of the model representation in all stages.

### Fitting of category-cluster RDMs to model and brain RDMs

Ten category-cluster RDMs (Figure S5) were created as predictors for a linear model of each RDM. The category clusters were: animate, inanimate, face, human face, non-human face, body, human body, non-human body, natural inanimate, and artificial inanimate. To measure the clustering strength for each of the categories in each brain and computational-model RDM, we fit the category-cluster RDMs to each brain and computational-model RDM minimizing the sum of squared dissimilarity deviations (Figure 3).

The design matrix for the least-squares fitting was created using the ten category RDMs (each RDM was vectorized to form a column in the design matrix) with addition of a constant vector of 1 (confound mean RDM). Then the category model RDMs were fitted to object-vision model RDMs. Bars in Figure S6 show the fitted coefficients (Beta values). Standard errors and p values are based on bootstrapping of the stimulus set. For each bootstrap sample of the stimulus set, a new instance is generated for the reference RDM (e.g. hIT RDM) and for each of the candidate RDMs (e.g. model RDMs). We did stratified resampling, which means that the proportion of categories was the same across all bootstrapped resamples. Because bootstrap resampling is resampling with replacement, the same condition can appear multiple times in a sample. This entails 0 entries (from the diagonal of the original RDM) in off-diagonal positions of the RDM for a bootstrap sample. These zeros are treated as missing values and excluded from the dissimilarities, across which the RDM correlations are computed. The number of bootstrap resamplings used in bootstrap tests was 10,000.

### Weighting model features

#### Remixing of features

For the not-strongly-supervised models as well as the deep supervised model representations, we attempted to create new features as linear combinations of the original features that specifically emphasize the missing categorical divisions. For the not-strongly-supervised models, we used combi27 features to find these linear combinations. Three linear support vector machine (SVM) classifiers for body/nonbody, face/non-face, and animate/inanimate categorization were trained on a set of 884 labeled images of isolated objects nonoverlapping with the set of 96 images. We then used the decision-value outputs of the classifiers as new features. The resulting single-feature RDMs for the not-strongly-supervised models are shown in Figure 5, top – one RDM for each SVM. For the deep supervised model, we used features from layer 7 to find linear combinations that emphasize the categorical divisions. The resulting single-feature RDMs for the deep supervised model are shown in Figure 10, top.

#### Reweighting of features

We tested whether appropriate weighting of the combination of the original model features and the new features learned by remixing could further improve the explanation of the IT geometry. We did the reweighting for both not-strongly-supervised model features, and deep supervised model features. For the not-strongly-supervised models, in addition to the 27 not-strongly-supervised models, we included the combi27 model and the three categorical SVM discriminants (learned through remixing) in the set of representations to be combined. We fitted one weight for each of these representations (27+1+3 = 31 weights in total), so as to best explain the hIT RDM. Figure 5, middle row, shows the weights obtained for each of the model representations. For the deep supervised model representations, we weighted the combination of all eight layers of the deep convolutional network and the three categorical SVM discriminants obtained by remixing the deep supervised features. Please note that one weight is learned for each layer, and each of the SVM discriminants (8+3 = 11 weights in total). Figure 10, middle row, shows the weights obtained for each of the layers of the deep convolutional network and the SVM discriminants.

We used a non-negative-least-squares fitting algorithm [51] to find the non-negative weights for the models that minimize the sum of squared deviations between the hIT RDM and the RDM of the weighted combination of models.

The RDM of the weighted combination of the model features is equivalent to a weighted combination of the RDMs of the models when squared Euclidean distance is used. We used the squared Euclidean distance for normalized representational patterns, which is equivalent to correlation distance, as used throughout this paper. We therefore applied the nonnegative least-squares algorithm at the level of the RDMs. This procedure is further explained in the following equations: equations (1) and (2).

Equation (1) states that the squared distance between weighted model features, equals the weighted squared distance of the features:
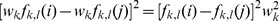
(1)Where *w_k_* is the weight given to model *k*. *f_k,l_(i)* is the *l^th^* feature extracted by model *k* for stimulus *i*.

Equation (2) shows how each of the *n* model representations are weighted by minimizing the sum of squared deviations between the hIT RDM and the RDM of the weighted combination of model representations.
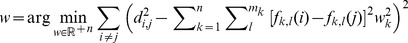
(2)Where *d_i,j_* is the distance between stimuli *i,j* in the hIT RDM. **w** is the weight vector that minimizes the sum of squared errors between the pairwise dissimilarities of the stimuli in the hIT representation and the pairwise dissimilarities of the weighted combination of model features. *k* changes from 1 to *n* where *n* is the number of model representations to be weighted. *m_k_* indicates the number of features for model *k*.

To avoid overfitting to the image set, we fitted the weights to random subsets of 88 of the 96 images in a crossvalidation procedure, holding out 8 images on each fold. The representational dissimilarities for the weighted-combination model was then estimated for the 8 held-out images. This procedure was repeated until the pairwise dissimilarities for the entire RDM of 96 by 96 images were estimated.

### IT-geometry model

The IT-geometry supervised models (i.e. IT-geometry-supervised combi27, and IT-geometry-supervised deep convolutional network) are made by remixing and reweighting of the model features. For the IT-geometry-supervised combi27, only the not-strongly-supervised models were used for remixing and reweighting; and for the IT-geometry-supervised deep convolutional network, only deep supervised model representations were used for remixing and reweighting.

For both of them, as explained before in the context of remixing and reweighting, we trained three SVM classifiers for animate/inanimate, face/nonface, and body/nonbody classification using 884 training images. The SVM classifiers were then fed with the 96 stimuli and we used the SVM decision values as new features. The non-negative least square fitting was then used for finding the optimal weights for different model representations and the SVM discriminant features so as to minimize the sum of squared errors between the RDM of the weighted combination of the features and the hIT RDM.

For making the IT-geometry supervised RDM, which is a weighted combination of the model representations and the SVM discriminants, we fit the non-negative weights by cross-validating the stimulus set. Each time we randomly left out 8 stimuli (4 animates and 4 inanimates) from the set of 96, and learned the optimal weights over the remaining stimuli (88 images) so as to minimize the sum of squared errors between the RDM of the weighted combination of the features and the hIT RDM. Note that the hIT RDM and the model RDMs become 88×88 (not 96×96) because 8 stimuli are left out. The obtained weights were then applied to weight the model feature for the left-out stimuli. The result is an 8×8 weighted RDM that shows the pairwise dissimilarities for the left-out stimuli. This procedure was repeated for several times until a point that we had the cross-validated pairwise dissimilarities for all the 96 stimuli.

### Categorization performance of models

We calculated the categorization performance of the object-vision models in the following categorization tasks: animates vs. inanimates (Figure 11), faces vs. bodies (Figure S11B), and artificial inanimates vs. natural inanimates (Figure S11A). For each of the models, a SVM classifier [70] with a linear kernel was trained using k-fold cross validation (k = 12). The 96 stimuli were randomly partitioned into k = 12 equal size folds. Of the *k* folds, a single fold was retained as the validation data for testing the model categorization performance, and the remaining *k−1* folds were used as training data. The cross-validation process was then repeated *k* times, with each of the *k* folds used exactly once as the validation data. The *k* results from the folds were then averaged.

For each of the categorization tasks the SVM was trained in the following way:

- For the animate vs. inanimate categorization task, we had 96 stimuli. We left out 8 stimuli (4 animates and 4 inanimates) that were used as the validation data, and the SVM was trained using the remaining stimuli.
- For the face vs. body categorization task, we had 48 stimuli. We left out 4 stimuli (2 faces and 2 bodies) that were used as the validation data, and the SVM was trained using the remaining stimuli.
- For the artificial vs. natural inanimate categorization task, again we had 48 stimuli. We left out 4 stimuli (2 artificial and 2 natural inanimates) that were used as the validation data, and the SVM was trained using the remaining stimuli.

To see if a model categorization performance significantly differs from chance, we did a permutation test by retraining the models after category-orthogonalized permutation of labels.

### Representational similarity analysis (RSA)

RSA enables us to relate representations obtained from different modalities (e.g. computational models and fMRI patterns) by comparing the dissimilarity patterns of the representations. In this framework representational dissimilarity matrices (RDMs) are used for making the link between different modalities. RDM is a square symmetric matrix in which the diagonal entries reflect comparisons between identical stimuli and are 0, by definition. Each off-diagonal value indicates the dissimilarity between the activity patterns associated with two different stimuli. RDM summarizes the information carried by a given representation from an area in the brain or a computational model.

We had 96 stimuli, of which half were animates and the other half were inanimates. To calculate the RDM for a brain region or a computational model, a 96×96 matrix was made in which each cell was filled with the dissimilarity value between the response patterns elicited by two stimuli. For each pair of stimuli, the dissimilarity measure was 1 minus the Pearson correlation between the response patterns elicited by those stimuli in a brain region or a computational model.

### Kendall *τ_A_* (tau-a) correlation and noise ceiling

To judge the ability of a model RDM in explaining a brain RDM, we used Kendall's rank correlation coefficient *τ*_A_ (which is the proportion of pairs of values that are consistently ordered in both variables). When comparing models that predict tied ranks (e.g. category model RDMs) to models that make more detailed predictions (e.g. brain RDMs, object-vision model RDMs) Kendall's *τ*_A_ correlation is recommended. In these occasions τA correlation is more likely than the Pearson and Spearman correlation coefficients to prefer the true model over a simplified model that predicts tied ranks for a subset of pairs of dissimilarities. For more information in this regard please refer to the RSA Toolbox paper [30]. This is the first toolbox to implement RSA. It is a modular and work-flow based toolbox that supports an analysis approach that is simultaneously data- and hypothesis-driven. There are a set of “Recipe” functions in the toolbox that allow automatic ROI analysis as well as whole-brain searchlight analysis. Tools for visualization and inference enable the user to relate sets of models to sets of brain regions and to statistically test and compare the models using nonparametric inference methods.

Figure 2 shows *τ*_A_ correlation of the hIT/mIT RDM with model RDMs. To estimate significance, randomization and bootstrap tests were used. Randomization tests permute the stimulus labels whereas bootstrap tests bootstrap resample the conditions set.

The noise in the brain activity data has imposed limitations on the amount of dissimilarity variance that a model RDM can explain. Therefore an estimation of noise-ceiling was needed to indicate how much variance of a brain RDM –given the noise level– was expected to be explained by an ideal model RDM (i.e. a model RDM that is able to perfectly capture the true dissimilarity structure of the brain RDM).

The noise-ceiling in Figure 2A is shown by a gray horizontal bar. The upper and lower edges of this bar correspond to upper- and lower-bound estimates on the group-average correlation with the RDM predicted by the unknown true model. There is a hard upper limit to the average correlation with the single-subject reference-RDM estimates that any RDM can achieve for a given data set. Intuitively, the RDM maximizing the group-average correlation lies at the center of the cloud of single-subject RDM estimates. To find an upper bound, we averaged the rank-transformed single-subject RDMs and used an iterative procedure to find the RDM that has the maximum average Kendall's *τ*_A_ correlation to the single-subject RDMs. This average RDM can be thought of as an estimate of the true model's RDM. This estimate is overfitted to the single-subject RDMs. Its average correlation with the latter therefore overestimates the true model's average correlation, thus providing an upper bound. To estimate a lower bound, we employed a leave-one-subject-out approach. We computed each single-subject RDM's correlation with the average of the other subjects' RDMs. This prevents overfitting and underestimates the true model's average correlation because the amount of data is limited, thus providing a lower bound on the ceiling. For more information about the noise ceiling please refer to the toolbox paper [30]. We did not estimate a noise ceiling for the cell recording data, because our procedure requires several individuals to be measured and we only had data for two monkeys.

### Equating the noise level in the models and the human IT

To compare the categoricality in the models with the categoricality in human IT, we added Gaussian noise to the models to equate the level of noise in the models with that of the fMRI data. To this end, we averaged the pairwise correlation between the IT RDMs of the four human subjects; let's denote the obtained value with ‘*q*’. Then to add the same amount of noise to the models, we iteratively and increasingly added noise to the model outputs until they reach the same level of noise as in human IT. The procedure for each model was that, we made new instantiations of that model by adding random Gaussian noise to the model output. We did this four times for each model, therefore having four noisy instantiation for each model. Then we made four model RDMs for each of the noisy model features, and calculated the mean of their pairwise correlation, which we denote by ‘*q_m_*’. If the obtained mean is equal to the mean of the pairwise correlation between the four hIT RDMs, denoted by ‘*q*’, (i.e. 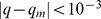) we stop the iteration, otherwise the procedure is repeated and in each iteration the added noise to the model output is updated. At the end, when the stopping criterion is satisfied, the four model RDMs are averaged and used as the noise-equated model RDM.

### Stimuli and response measurements

We used the experimental stimuli from Kriegeskorte et al. [7]. The stimuli were 96 images which half were animates and the other half were inanimates. The animate cluster consisted of faces and bodies, and the inanimate cluster consisted of natural and artificial inanimates.

For cell recording data, we had 92 stimuli. To make 92×92 RDMs comparable with 96×96 RDMs, we made a 96×96 RDM from 92×92 RDM by filling the gaps with NaN.

The fMRI and cell recording data, which we used here, have been previously described and analyzed to address different questions. See [6], [7], [18] for further experimental details.

## Discussion

Computer vision has made great strides in recent years. Early attempts to achieve vision by fitting generative graphics models to images faltered because of the exponential complexity of the search space. However, computer vision made progress in practical applications using hand-engineered feedforward features in combination with machine learning classifiers. In recent years, the advent of efficient training algorithms for deep neural networks [40], [71], [72], [41] has made it possible to learn from image data not just the final classification step, but also the internal representations. This approach has yielded unprecedented object-recognition performance, reaching levels comparable to humans on certain tasks (e.g. [41], [73]).

These new deep vision models share certain features, some of which parallel the primate visual system. First, they are feedforward hierarchical models: They are composed of a series of stages of representation, where each stage is computed from the output of the previous stage. Moderate modifications of this scheme with bypass connections are also sometimes used. Second, each stage is composed of features, which are linear filters of the previous stage followed by a static nonlinearity. The nonlinearity is key to the representational power of these networks because a sequence of linear transforms would reduce to a single linear transformation. Third, they are convolutional [40], computing each linear feature of the input at all visual-field locations. This architectural constraint reduces the effective number of parameters and automatically confers translation invariance. Fourth, they compute visually local features with receptive field sizes increasing from stage to stage, thus gradually transforming a space-based image-like representation into space-insensitive shape-based and semantic representation. Fifth, they are deep, typically including four or more layers of representation. Even shallow neural networks can approximate any nonlinear mapping from input to output. However, deep networks can find concise representations (requiring fewer units) of complex functions. This is essential to make them realistic in terms of both physical implementation and learnability [72]. Sixth, they are trained with many category-labeled example images, typically more than a million (e.g. [41]).

A few studies have begun to compare recognition performance and internal representations between these models and primate IT. These investigations have so far given largely convergent results. First, models that perform better at object recognition tend to have representations more similar to IT [74]–[76]. Second, the new deep supervised models perform at unprecedented levels at predicting the IT representation [75]–[77].

Our exploration here placed deep supervised models in the context of a wide range of computer-vision features, revealing the extent to which each of these computational mechanisms can explain the IT representational geometry in human and monkey. In addition, we analyzed the degree to which each of the models emphasizes various categorical divisions. Our results, spanning the gamut from unsupervised to strongly supervised models, suggest that strong supervision with many category-labeled images is essential for building features that explain the IT representational geometry. The not-strongly-supervised models were significantly less categorical than IT and this was part of the reason why they failed to explain the IT representational geometry. In addition, IT appears to have a particular categorical geometry. This is consistent with the idea that IT is visuo-semantic, representing visual features including shape, but also imposing categorical divisions (or emphasizing semantic dimensions) that are relevant to the organism's survival and reproduction.

We find strong similarities between the representational geometries of a deep supervised model and IT (see also [76]). This is important because it suggests that deep supervised models capture something essential about the IT representation. However, the fact that our IT-geometry-supervised deep representation fully explains our IT data should not be overinterpreted.

First, these models operate in a feedforward fashion, and do not capture the recurrent dynamics in the visual hierarchy. This component of visual processing might be sufficient for “core object recognition” [78], i.e. rapid recognition at a glance. However, vision provides us with a much more complex appreciation of our surroundings and supports a wide array of tasks. Biological vision involves recurrent processing as well as active exploration of the scene with attentional shifts and eye movements. In the present experiments, stimuli were presented for 105 ms (monkeys) and 300 ms (humans) and eye movements and object-related attentional processes were minimized by using fixation tasks. The experiments were, thus, designed to focus on automatic, task-independent processing. However, recurrent processing is nevertheless likely to have contributed to the emergence of the IT representation. Indeed, recent human magnetoencephalography studies using the same stimulus set [11], [12] suggest that the major categorical divisions take slightly longer to emerge than a purely feedforward account would predict. This evidence is not unequivocally localized to IT and thus should be interpreted with caution. However, the categorical clustering achieved in a purely feedforward fashion in the deep supervised model considered here might be achieved with some degree of involvement of recurrent computations in the brain.

Second, the IT-geometry-supervised model needed to be explicitly trained to emphasize the same categorical divisions as IT. The analysis of our human and monkey data in [77] similarly found that a hierarchical feedforward model optimized for invariant object recognition could account for the IT representational geometry only when linear readout features emphasizing the appropriate categorical divisions were fitted to the data. On the one hand, our study suggests that visual similarity (as operationalized by the wide range of unsupervised visual features we investigated) cannot explain the categorical clustering. On the other hand, it begs the question why IT emphasizes the particular divisions between faces and bodies and between animates and inanimates, while deemphasizing other divisions (such as the one between human and animal faces).

Third, our study is limited by the image set. All objects were centered on fixation and presented in isolation on a gray background at the same retinal size. The stimulus set, thus, was not challenging in terms of position, size, and clutter invariance. However, the IT representation has been shown to be less sensitive to changes of position, size, and context than earlier stages of processing [79]. Cadieu et al. (2014) [76] used an image set with substantial variations of position, size, and clutter to compare the representation in the same deep supervised model to monkey-IT data and found the categorical clustering to be robust to these variations. Although our image set did not vary position, size, and clutter, note that it covered a broad range of categories and within each category, there was substantial variation among the exemplars in terms of both their intrinsic properties and accidental properties of their appearance, including pose and lighting. The wide exemplar variations within broad categories like animates might present an even more difficult challenge than varying position and size. The human face photos were mostly frontal and therefore visually similar (as reflected in the clustering of human faces in many of the unsupervised feature models). However, the variation among the animal faces and among the exemplars within the animate and inanimate categories was substantial. Taken together, current evidence suggests that the categorical clustering we observed is not an artefact of the stimulus set.

Finally, our data set was affected by noise and intersubject variation. The human fMRI data, for which we were able to compute the noise ceiling, was from 8 sessions (2 sessions in each of four subjects [7]). The fact that the IT-geometry-supervised deep model fully explained the representational geometry of IT does not mean that its representation is identical to IT, but just that given noise and intersubject variability it is not significantly different. Future studies should use more comprehensive data sets to reveal remaining representational discrepancies between IT and deep supervised models.

### What does it mean for a representation to be “categorical” or “semantic”?

The IT representation has been described as categorical by some authors [7] and as a visual shape space by others [23]. How should a “categorical” representation be defined in this context?

One meaning of categoricality refers to the degree to which categorical divisions are *explicit* in the representation. The images themselves (and their retinal representations) clearly contain category information. However, this information is not explicit. Instead it requires a highly nonlinear readout mechanism commonly referred to as object recognition. An explicit representation is sometimes defined [78], [80] as one that enables linear readout of the category dichotomy. Since linear readout is a trivial one-step operation in a biological neuronal network, this definition of “explicit” is arguably only slightly broader than requiring single-cell step-like responses encoding the category dichotomy. Linear discriminability does not require that the categories form separate clusters in the representational space. A bimodal distribution in representational space, with two clusters corresponding to the categories and divided by a margin or region of lower density, could be considered to be an even more explicitly categorical representation than one that merely enabled linear readout.

Defining categoricality as the degree to which category information is explicit (as all the above definitions do) may be useful in some contexts. However, it misses a crucial point. Depending on the nature of the images and categories, “explicit” category representations could be observed in: (1) pixel images or color histograms, (2) simple computational features (e.g. Gabor filters or gist features), (3) more complex unsupervised features (e.g. HMAX features). If the features happen to be sufficiently correlated with a categorical division, these “visual” representations would be considered explicitly categorical by the above definitions. This illustrates the difficulty of drawing a clear line between visual and categorical (or semantic) representations.

We would rather not refer to the representation as “categorical” when the categories are already separated in the distribution of the sensory input patterns. We therefore suggest a criterion distinct from category explicitness as the defining property of a categorical representation. A representation is “categorical” when it affords *better* category discriminability than any feature set that can be learned without category supervision, i.e. when it is *designed* to emphasize categorical divisions. A categorical representation in this sense can be interpreted as serving the function to emphasize behaviorally relevant categorical divisions or semantic dimensions.

A category is a discrete semantic variable. A semantic representation could also include continuous variables that describe visual objects. Categorical clusters in the representational space do not require discrete categorical variables. A sufficient prevalence of continuous semantic variables that are correlated with a given categorical division could also produce categorical clusters. Future studies should investigate in greater detail whether the semantic component of the IT representation is better accounted for by categorical or continuous semantic dimensions.

### The IT representation appears to be both visual and semantic

Several studies suggested that the IT representation is not purely visual but also semantic [7], [14], [22], [81]. Our study provides additional support for this claim by showing that IT exhibits significantly stronger category clustering than a wide range of unsupervised models. It is impossible to prove that no visual feature model built without category-label supervision can explain the IT representation. However, our current interpretation is that IT reflects knowledge of category boundaries or semantic dimensions, and is thus not purely visual.

This finding may appear to contradict a previous study suggesting that the IT representation is better accounted for by visual shape than by semantic category [23]. Note, however, that the representation of visual shape in IT is uncontroversial. A better account on the basis of visual shape does not preclude an additional semantic component. There is clearly a continuum between visual and semantic, between the representation of the appearance and the representation of the behavioral significance of an object. Our working hypothesis is that the function of the primate ventral stream is to achieve this transformation. Intermediate-level features detecting parts of objects (e.g. eyes, noses, ears) might provide a stepping stone toward semantics and could lead to clustering of faces and animates [82], [83].

Recognition requires abstracting from several sources of within-category variation among object images. One source of variation lies in the accidental properties [84] of the appearance of the object, such as its pose, distance, and lighting. Another source of within-category variation are the substantial differences between exemplars. In our study, the winning model was supervised with category-labeled images, learning to abstract from both of these sources of variation. It would be interesting to investigate whether training a representation to abstract from accidental properties only with exemplar-label supervision (where multiple images of the same particular object have the same exemplar label) can also produce a representation similar to IT. To our knowledge, however, the previous studies [75], [76] that investigated accidental property variation in greater detail also required category-label supervision to derive representational geometries resembling that of IT.

### How do biological brains acquire categorical divisions?

In this study, we were looking to discover a model of the mechanism of biological object vision. We did not attempt to model the developmental process that builds that mechanism. Creating a viable model of IT appeared to require supervised learning. How might biological development implement this process? Biologically plausible implementations of backpropagation and related rules for supervised learning have been proposed (e.g. [85]). However, it is unclear what supervision signal such a process would use. What is the equivalent of the category labels in the biological development of the IT representation? One possibility is that the perceptual and behavioral context provides the equivalent of the supervision signal in natural development.

For example, visual images appearing in the same temporal context will often represent the same object in different retinal positions, poses, distances, and sizes. It has been argued that invariance to accidental properties can be learned from temporal proximity in natural experience [86]–[88]. Different visual images in the same temporal context will also tend to represent the same scene. A biological learning mechanism that associates visual inputs that tend to co-occur with similar representational patterns would learn features that are more stable across time, abstracting from rapidly changing aspects of visual appearance. Moreover, objects present in a given scene might tend to be semantically related. Such a mechanism might therefore even learn semantic features.

Another way that context might provide a stepping stone toward a semantic representation is through perceptual channels beyond the current retinal image. Natural perception provides a rich multimodal and dynamic stream of information. Distinct visual patterns associated with similar context percepts might come to be represented together in the representational space. For example, visual motion is associated with animacy [89], so dissimilar shapes associated with the same visual motion patterns might come to be co-located in the representational space.

The argument from context can be extended to other sensory modalities (e.g. the same sound associated with two distinct visual stimuli), and to behavioral and social context, which might contain signals correlated with the categories of the objects present in the scene [90]. Visually dissimilar stimuli may be associated with the same linguistic utterances of contemporaries, or with the same physical actions [91] or emotional states. Finally, the cognitive context, including conscious inferences based on our perception of the current scene and behavioral goals, might influence the development of the IT representation through feedback signals from frontal regions that provide an endogenous context to natural visual experience.

An unsupervised learning process that receives such context signals alongside the visual input would be expected to cluster percepts that are similar in this more complex multimodal input space. The resulting representational clusters might then persist when the context is removed from the input and only static visual shapes are presented, as in our experiments. The argument from context illustrates how the distinction between supervised and unsupervised learning, which is clearly defined in computer science, is blurred for biological brains. Unsupervised learning from a richly contextualized sensory input might achieve a result similar to that of supervised learning.

### Explaining the IT representation requires considering what it is for

The ultimate purpose of vision is not to provide a veridical representation of our visual environment, but to support successful behavior. An explanation of the IT representation, then, requires consideration of behavioral affordances. It appears plausible that any primate faced with an unknown object might want to determine whether it is animate with high priority. Similarly, faces are important to recognize because they confer a host of information that renders animates somewhat more predictable. In computational modelling, such behavioral affordances can be brought in by optimizing the representations for particular categorization tasks, using supervised training. Such task-specific performance optimization appears essential to explaining IT. Models with higher recognition accuracy better explained not only the categorical clusters, but also the within-category representational geometries observed in IT.

Our results suggest that the IT representation is visuo-semantic. Explaining IT requires consideration of the perceptual and cognitive context and of behavioral affordances. Through phylo- and ontogenesis, IT appears to have learned to emphasize certain behaviorally important divisions that transcend visual appearance and relate to the meaning of objects in the context of the organism's survival and reproduction.

## Supporting Information

Figure S1 **The not-strongly-supervised models best explaining EVC (A), FFA (B), LOC (C), and PPA (D).** This figure shows the most correlated model RDMs (from left to right and top to bottom) with the EVC (A), FFA (B), LOC (C) and PPA (D) RDMs. Biologically motivated models are set in black font, and computer-vision models are set in gray font. Models with the subscript ‘UT’ are unsupervised trained models; and others without a subscript are untrained models. The number below each RDM is the Kendall τ_A_ correlation coefficient between the model RDM and the respective brain RDM. All correlations are statistically significant, except those that are shown by ‘ns’. Correlation p-values are reported in Table 1.
(TIF)

Figure S2 **Kendall's τ_A_ RDM correlation of the not-strongly-supervised models with EVC (A), FFA (B), LOC (C), and PPA (D).** The bars shows the Kendall's *τ*_A_ RDM correlation between the not-strongly-supervised model RDMs and EVC (A), FFA (B), LOC (C) and PPA (D). The error bars are standard errors of the mean estimated by bootstrap resampling. Asterisks across the x-axis show the p-values obtained by a random permutation test based on 10,000 randomizations of the condition labels (ns: not significant, p<0.05: *, p<0.01: **, p<0.001: ***, p<0.0001: ****). These p-values assess the relatedness of different model RDMs with a brain RDM. The grey horizontal rectangle shows the noise ceiling.
(TIF)

Figure S3 **Kendall's τ_A_ RDM correlation of the deep convolutional network with EVC (A), FFA (B), LOC (C), and PPA (D).** The bars show the Kendall-τ_A_ RDM correlations between the layers of the deep supervised convolutional network and EVC (A), FFA (B), LOC (C) and PPA (D). The error bars are standard errors of the mean estimated by bootstrap resampling. Asterisks across the x-axis show the p-values obtained by a random permutation test based on 10,000 randomizations of the condition labels (ns: not significant, p<0.05: *, p<0.01: **, p<0.001: ***, p<0.0001: ****). The grey horizontal rectangles show the noise ceiling in each of the brain ROIs. The upper and lower edges of the gray horizontal bar are upper and lower bound estimates of the maximum correlation any model can achieve given the noise.
(TIF)

Figure S4 **Different combinations of the not-strongly-supervised models.** Each of the first four RDMs (A, B, C, D) was calculated by combining internal representation of object-vision models for all images and then measuring the pairwise dissimilarity between the combined feature vectors. E and F are categorical model RDMs; F shows animate-inanimate category structure, and E comes with extra information about the within-animate category structure (i.e. face clusters). Underneath each RDM, the Kendall-τ_A_ correlations of that RDM with hIT and mIT RDMs are stated. The statistical significance of correlations are shown by asterisks (p<0.05: *, p<0.01: **, p<0.001: ***, p<0.0001: ****). To estimate significance, randomization test was used.
(TIF)

Figure S5 **Ten category RDMs used as linear predictors in the RDM model.** These ten category models and a confound mean (all-1) RDM were linearly combined to explain each of the brain and model RDMs (Figures 3, 4).
(TIF)

Figure S6 **Clustering strength for different categories in IT and not-strongly-supervised models.** We measured the strength of clustering for each of the categories (animate, inanimate, face, human face, non-human face, body, human body, non-human body, natural inanimates, and artificial inanimates), by least-squares fitting of a set of category cluster RDMs (shown in Figure S5) to each brain and computational-model RDM. Bars in this figure show the fitted coefficients (clustering strengths). The higher the bar, the more tightly clustered are the objects in that category. Error bars show 95% confidence interval of the coefficient estimates. Significance is shown by red (legend) corrected for 30 * 10 multiple comparisons. Standard errors and p values are based on bootstrapping of the stimulus set.
(TIF)

Figure S7 **Category-clustering strengths of not-strongly-supervised models relative to hIT.** For each of the categories (animate, inanimate, face, human face, non-human face, body, human body, non-human body, natural inanimates, and artificial inanimates) the difference in clustering strength between the models and hIT was measured. Bars show the difference in clustering strength between the models and hIT. Model clustering strengths that were significantly lower/higher than the hIT clustering strength are shown by blue/red bars (legend). Error bars show 95% confidence interval of the difference in clustering strength estimates between the models and hIT. P values are based on bootstrapping of the stimulus set.
(TIF)

Figure S8 **Category-clustering strengths of not-strongly-supervised models relative to mIT.** For each of the categories (animate, inanimate, face, human face, non-human face, body, human body, non-human body, natural inanimates, and artificial inanimates) the difference in clustering strength between the models and mIT was measured. Bars show the difference in clustering strength between the models and mIT. Model clustering strengths that were significantly lower/higher than the mIT clustering strength are shown by blue/red bars (legend). Error bars show 95% confidence interval of the difference in clustering strength estimates between the models and mIT. P values are based on bootstrapping of the stimulus set.
(TIF)

Figure S9 **Categoricality in noise-less models compared with the categoricality in IT.** Bars show categoricality (measured by the category clustering index, CCI) for each of the not-strongly-supervised models.The category clustering index (CCI) for each model and brain RDM is defined as the proportion of RDM variance explained by the category cluster model (Figure S5), i.e. the squared correlation between the fitted category-cluster model and the RDM it is fitted to. Error bars and shaded regions indicate 95%-confidence intervals. Significant CCIs are indicated by stars underneath the bars (* p<0.05, ** p<0.01, *** p<0.001, **** p<0.0001). Significant differences between the CCI of each model and the hIT/mIT CCI are indicated by blue/gray vertical arrows (p<0.05, Bonferroni-adjusted for 28 tests). The corresponding inferential comparisons for mIT are indicated by gray vertical arrows. The categoricality in hIT is significantly higher than in any of the 28 not-strongly-supervised models. This analysis is based on the noise-less model representations.
(TIF)

Figure S10 **Categoricality in the noise-less representations of the deep convolutional network compared with hIT and mIT.** Bars show categoricality (measured by the category clustering index, CCI) for each layer of the deep convolutional network and for the IT-geometry-supervised layer. For conventions and for definition of the CCI, see Figure S9. Error bars and shaded regions indicate 95%-confidence intervals. Significant CCIs are indicated by stars underneath the bars (* p<0.05, ** p<0.01, *** p<0.001, **** p<0.0001). Significant differences between the CCI of each model and the hIT/mIT CCI are indicated by blue/gray vertical arrows (p<0.05, Bonferroni-adjusted for 9 tests). The corresponding inferential comparisons for mIT are indicated by gray vertical arrows. Categoricality is significantly greater in hIT and mIT than in any of the internal layers of the deep convolutional network. However, the IT-geometry-supervised layer (remixed and reweighted) achieves a categoricality similar to IT. This analysis is based on the noise-less model representations.
(TIF)

Figure S11 **Categorization accuracy of all models for natural/artificial (A) and face/body (B).** Each dark blue bar shows the categorization accuracy of a linear SVM applied to one of the computational model representations. Categorization accuracy for each model was estimated by 12-fold crossvalidation on the 96 stimuli. To assess whether categorization accuracy was above chance level, we performed a permutation test, in which we retrained the SVMs on (category-orthogonalized) 10,000 random dichotomies among the stimuli. Light blue bars show the average model categorization accuracy for random label permutations. Categorization performance was significantly greater than chance for most models (ns: not significant, * p<0.05, ** p<0.01, *** p<0.001, **** p<0.0001).
(TIF)

Figure S12 **Kendall's** ***τ*** **_A_ RDM correlation of the not-strongly-supervised models with the hIT animate (A) and inanimate (B) sub-clusters.** The bars show the Kendall-τ_A_ RDM correlations of the not-strongly-supervised models with the hIT RDM for animate images (A), and inanimate images (B). The error bars are standard deviations of the mean estimated by bootstrap resampling. Asterisks across the x-axis show the p-values obtained by a random permutation test based on 10,000 randomizations of the condition labels (ns: not significant, p<0.05: *, p<0.01: **, p<0.001: ***, p<0.0001: ****). The p-values assess the relatedness of different model RDMs with a brain RDM. The grey horizontal rectangles show the noise ceiling. Models with the subscript ‘UT’ are unsupervised trained models, models with the subscript ‘ST’ are supervised trained models, and others without a subscript are untrained models.
(TIF)

Figure S13 **Kendall's** ***τ*** **_A_ RDM correlation of the not-strongly-supervised models with the mIT animate (A) and inanimate (B) sub-clusters.** The bars show the Kendall-τ_A_ RDM correlations of the not-strongly-supervised models with the mIT RDM for animate images (A), and inanimate images (B). The error bars are standard deviations of the mean estimated by bootstrap resampling. Asterisks across the x-axis show the p-values obtained by a random permutation test based on 10,000 randomizations of the condition labels (ns: not significant, p<0.05: *, p<0.01: **, p<0.001: ***, p<0.0001: ****). The p-values assess the relatedness of different model RDMs with a brain RDM. Models with the subscript ‘UT’ are unsupervised trained models, models with the subscript ‘ST’ are supervised trained models, and others without a subscript are untrained models.
(TIF)

Text S1 **Models better explain the representation of the animate objects than the inanimate objects in IT.**
(DOCX)
