# Supplementary figures and images for: Deep Supervised, but Not Unsupervised, Models May Explain IT Cortical Representation
Source: PLoS Comput Biol. 2014 Nov 6;10(11):e1003915. doi: 10.1371/journal.pcbi.1003915 (PMC4222664; doi:10.1371/journal.pcbi.1003915)

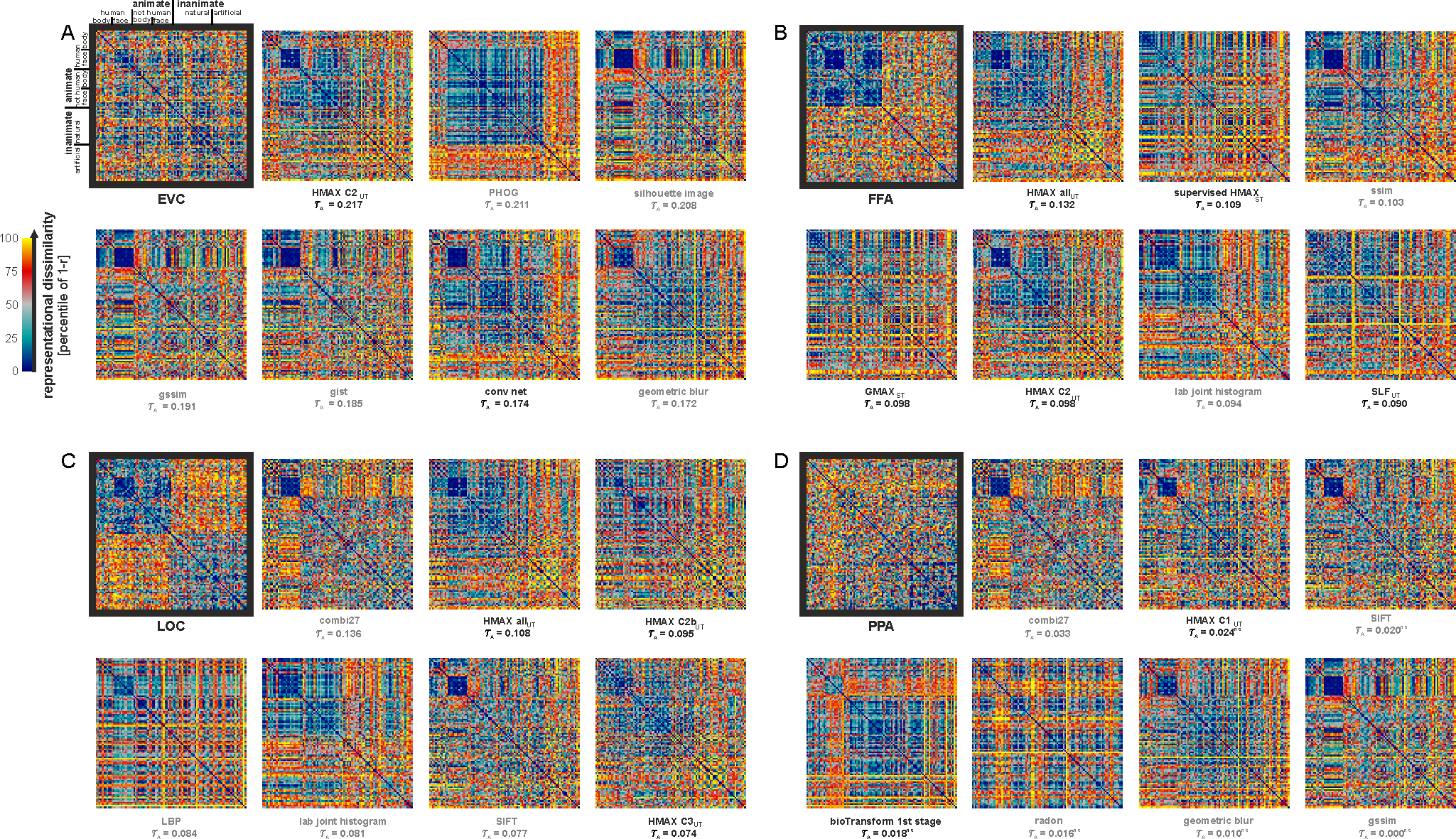

Supplement: Figure S1 — The not-strongly-supervised models best explaining EVC (A), FFA (B), LOC (C), and PPA (D). This figure shows the most correlated model RDMs (from left to right and top to bottom) with the EVC (A), FFA (B), LOC (C) and PPA (D) RDMs. Biologically motivated models are set in black font, and computer-vision models are set in gray font. Models with the subscript ‘UT’ are unsupervised trained models; and others without a subscript are untrained models. The number below each RDM is the Kendall τA correlation coefficient between the model RDM and the respective brain RDM. All correlations are statistically significant, except those that are shown by ‘ns’. Correlation p-values are reported in Table 1. (TIF) [file pcbi.1003915.s001.tif]

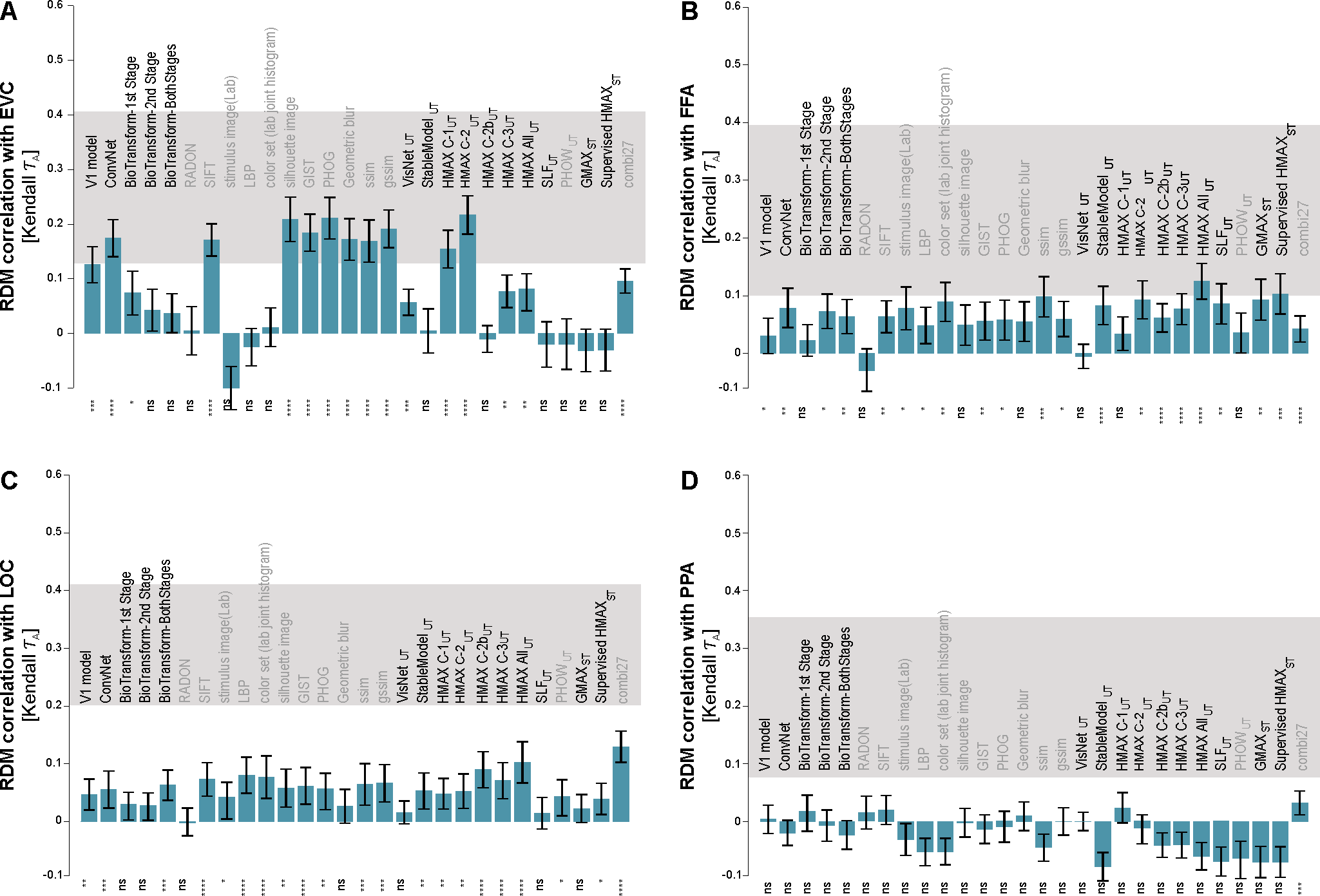

Supplement: Figure S2 — Kendall's τA RDM correlation of the not-strongly-supervised models with EVC (A), FFA (B), LOC (C), and PPA (D). The bars shows the Kendall's τ A RDM correlation between the not-strongly-supervised model RDMs and EVC (A), FFA (B), LOC (C) and PPA (D). The error bars are standard errors of the mean estimated by bootstrap resampling. Asterisks across the x-axis show the p-values obtained by a random permutation test based on 10,000 randomizations of the condition labels (ns: not significant, p<0.05: *, p<0.01: **, p<0.001: ***, p<0.0001: ****). These p-values assess the relatedness of different model RDMs with a brain RDM. The grey horizontal rectangle shows the noise ceiling. (TIF) [file pcbi.1003915.s002.tif]

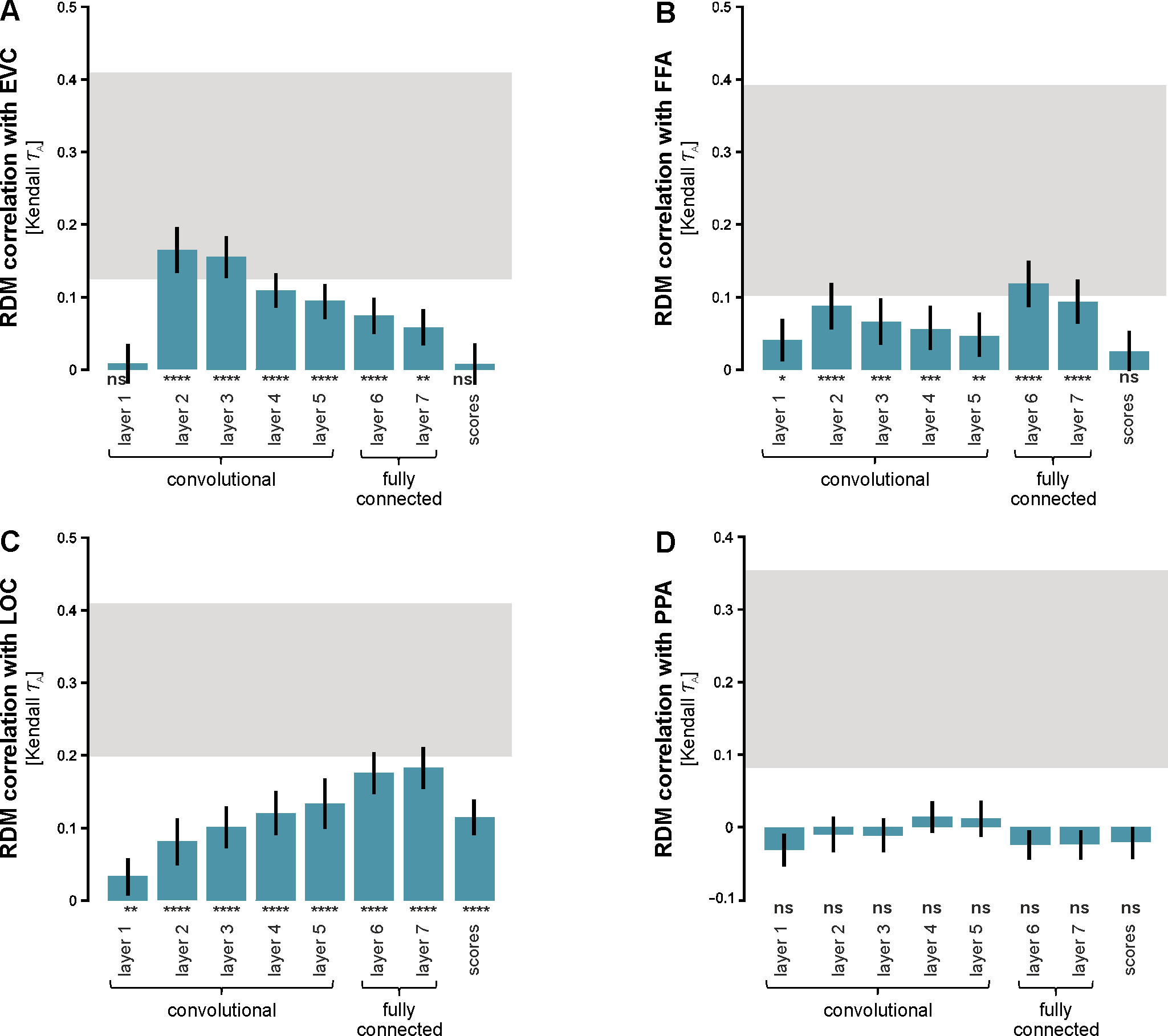

Supplement: Figure S3 — Kendall's τA RDM correlation of the deep convolutional network with EVC (A), FFA (B), LOC (C), and PPA (D). The bars show the Kendall-τA RDM correlations between the layers of the deep supervised convolutional network and EVC (A), FFA (B), LOC (C) and PPA (D). The error bars are standard errors of the mean estimated by bootstrap resampling. Asterisks across the x-axis show the p-values obtained by a random permutation test based on 10,000 randomizations of the condition labels (ns: not significant, p<0.05: *, p<0.01: **, p<0.001: ***, p<0.0001: ****). The grey horizontal rectangles show the noise ceiling in each of the brain ROIs. The upper and lower edges of the gray horizontal bar are upper and lower bound estimates of the maximum correlation any model can achieve given the noise. (TIF) [file pcbi.1003915.s003.tif]

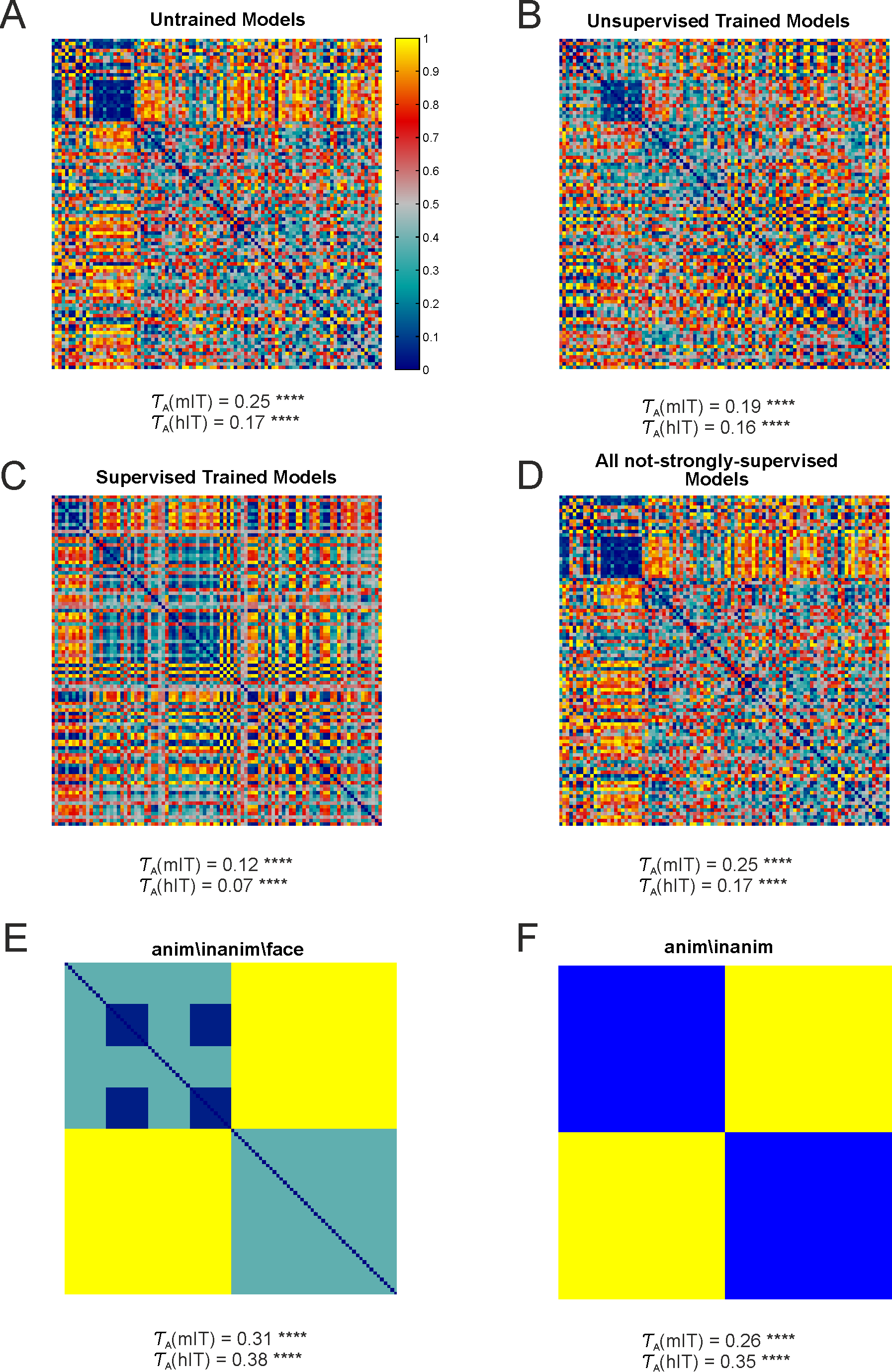

Supplement: Figure S4 — Different combinations of the not-strongly-supervised models. Each of the first four RDMs (A, B, C, D) was calculated by combining internal representation of object-vision models for all images and then measuring the pairwise dissimilarity between the combined feature vectors. E and F are categorical model RDMs; F shows animate-inanimate category structure, and E comes with extra information about the within-animate category structure (i.e. face clusters). Underneath each RDM, the Kendall-τA correlations of that RDM with hIT and mIT RDMs are stated. The statistical significance of correlations are shown by asterisks (p<0.05: *, p<0.01: **, p<0.001: ***, p<0.0001: ****). To estimate significance, randomization test was used. (TIF) [file pcbi.1003915.s004.tif]

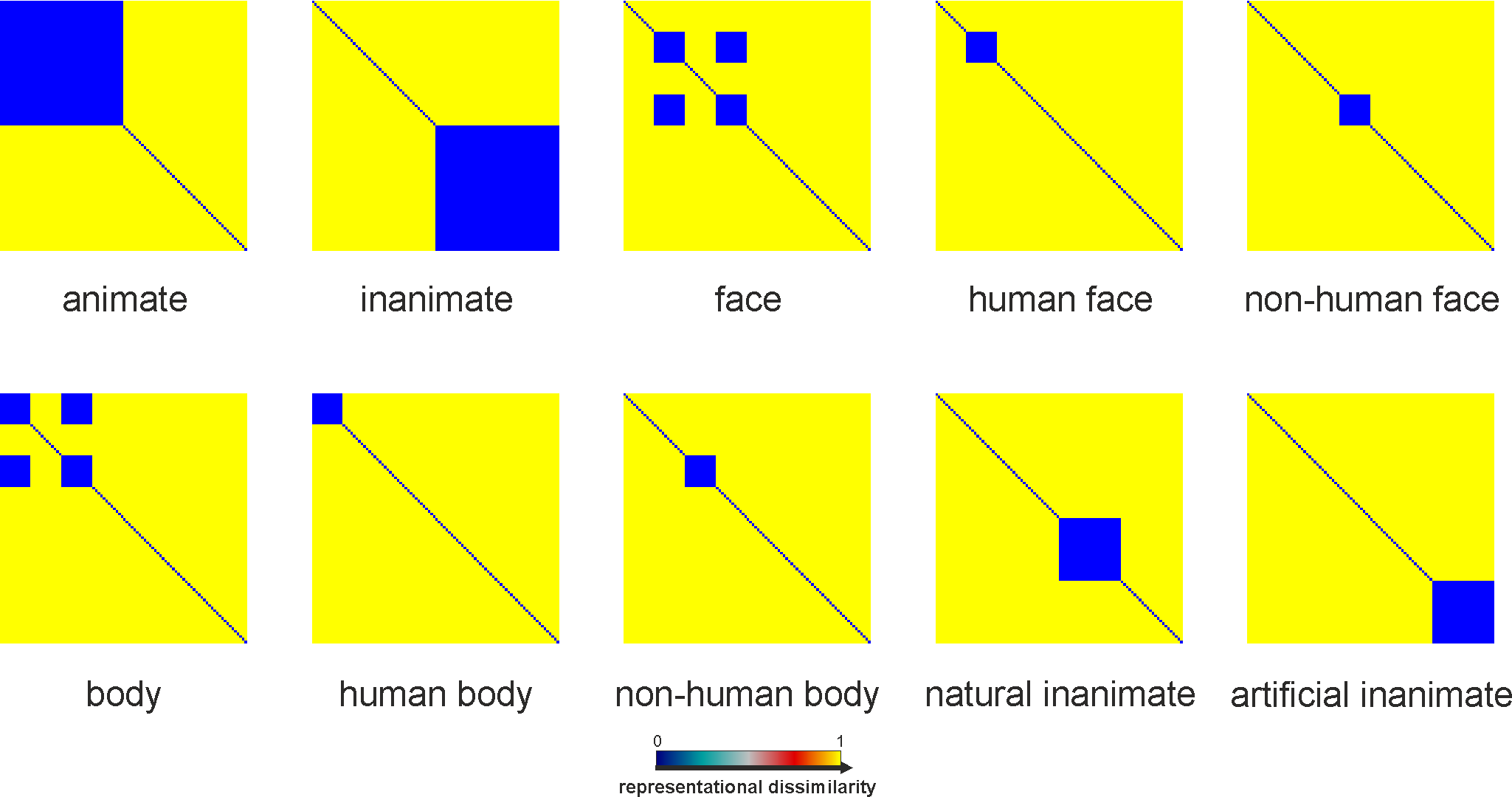

Supplement: Figure S5 — Ten category RDMs used as linear predictors in the RDM model. These ten category models and a confound mean (all-1) RDM were linearly combined to explain each of the brain and model RDMs (Figures 3, 4). (TIF) [file pcbi.1003915.s005.tif]

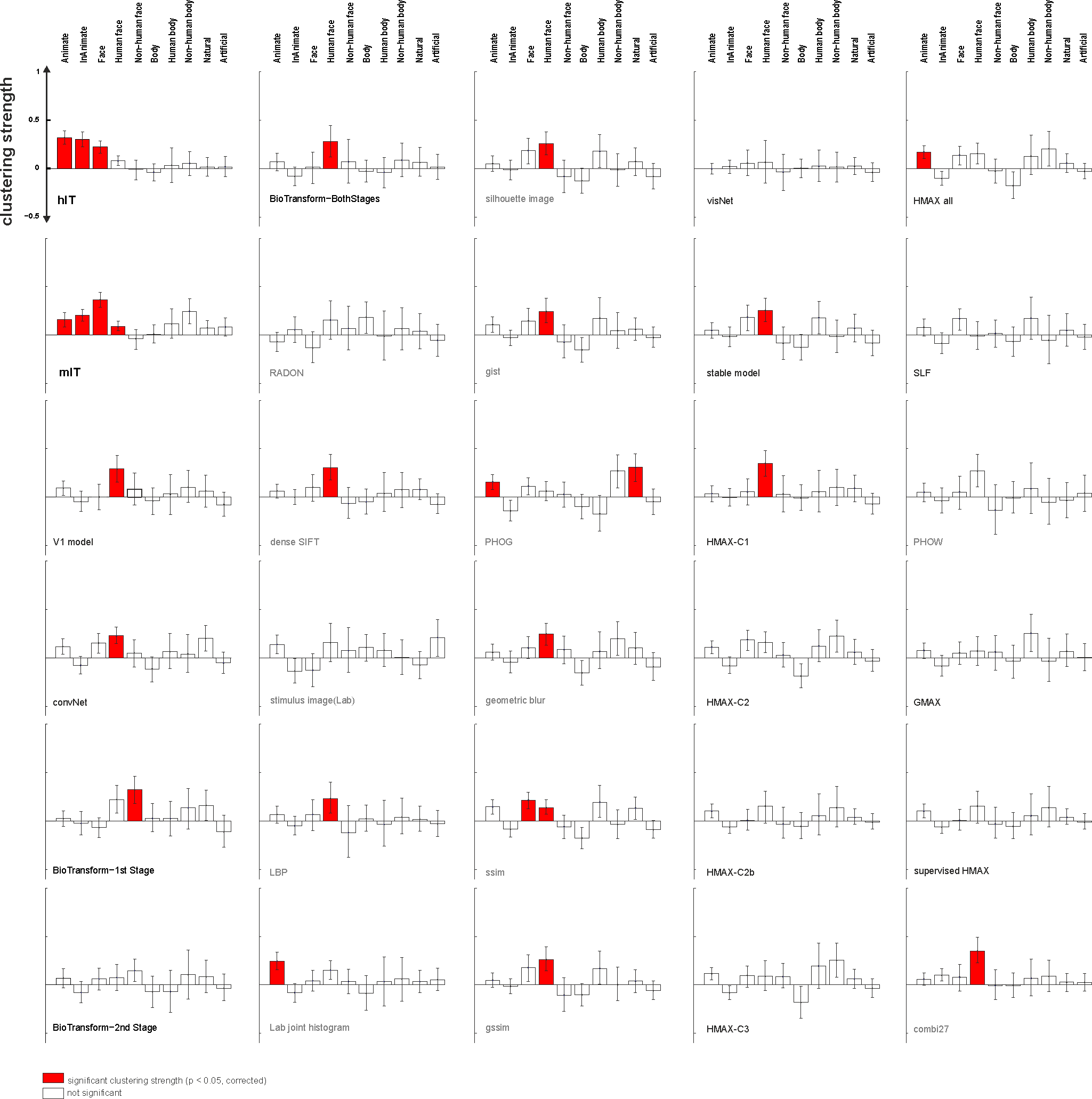

Supplement: Figure S6 — Clustering strength for different categories in IT and not-strongly-supervised models. We measured the strength of clustering for each of the categories (animate, inanimate, face, human face, non-human face, body, human body, non-human body, natural inanimates, and artificial inanimates), by least-squares fitting of a set of category cluster RDMs (shown in Figure S5) to each brain and computational-model RDM. Bars in this figure show the fitted coefficients (clustering strengths). The higher the bar, the more tightly clustered are the objects in that category. Error bars show 95% confidence interval of the coefficient estimates. Significance is shown by red (legend) corrected for 30 * 10 multiple comparisons. Standard errors and p values are based on bootstrapping of the stimulus set. (TIF) [file pcbi.1003915.s006.tif]

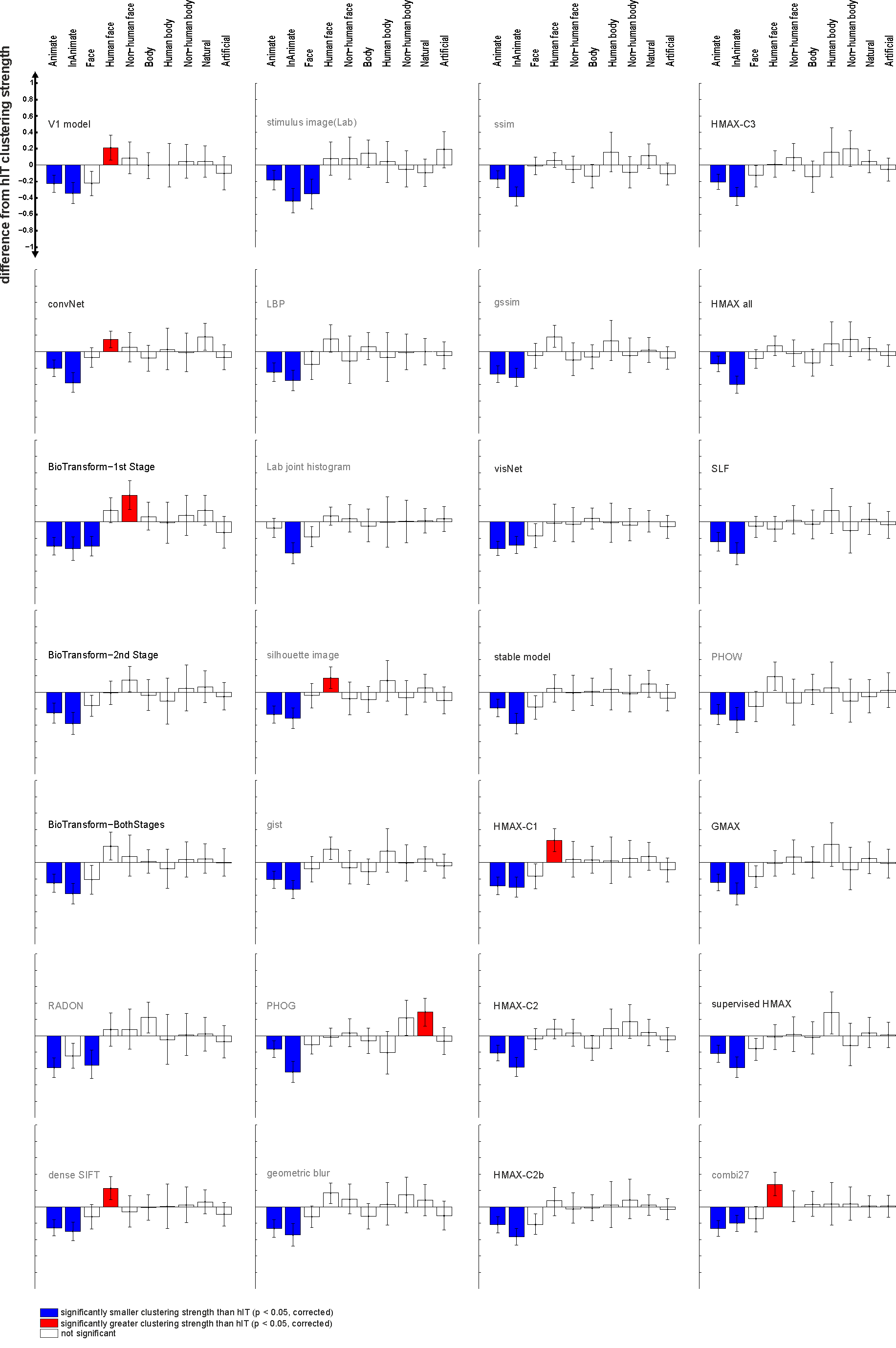

Supplement: Figure S7 — Category-clustering strengths of not-strongly-supervised models relative to hIT. For each of the categories (animate, inanimate, face, human face, non-human face, body, human body, non-human body, natural inanimates, and artificial inanimates) the difference in clustering strength between the models and hIT was measured. Bars show the difference in clustering strength between the models and hIT. Model clustering strengths that were significantly lower/higher than the hIT clustering strength are shown by blue/red bars (legend). Error bars show 95% confidence interval of the difference in clustering strength estimates between the models and hIT. P values are based on bootstrapping of the stimulus set. (TIF) [file pcbi.1003915.s007.tif]

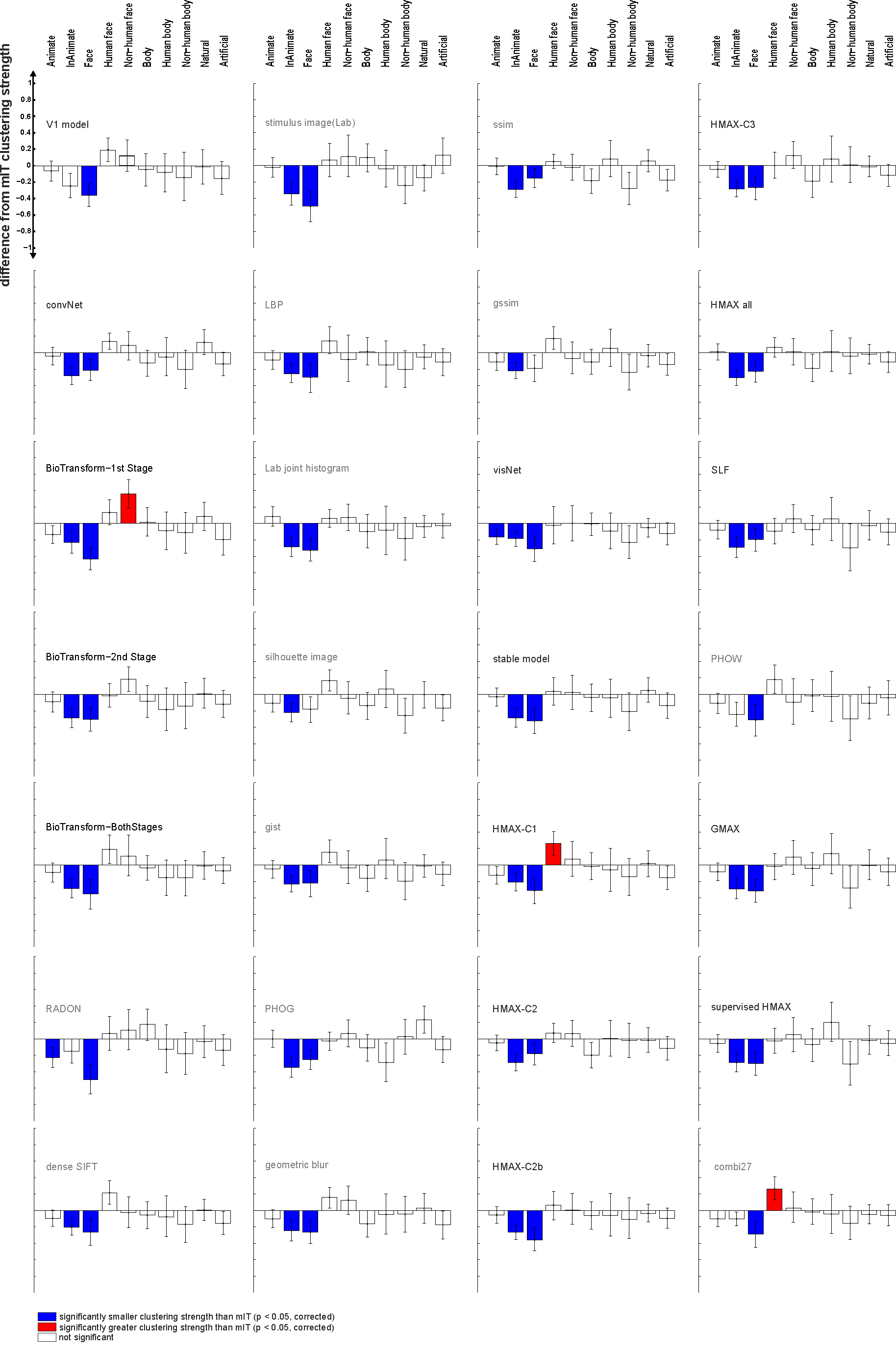

Supplement: Figure S8 — Category-clustering strengths of not-strongly-supervised models relative to mIT. For each of the categories (animate, inanimate, face, human face, non-human face, body, human body, non-human body, natural inanimates, and artificial inanimates) the difference in clustering strength between the models and mIT was measured. Bars show the difference in clustering strength between the models and mIT. Model clustering strengths that were significantly lower/higher than the mIT clustering strength are shown by blue/red bars (legend). Error bars show 95% confidence interval of the difference in clustering strength estimates between the models and mIT. P values are based on bootstrapping of the stimulus set. (TIF) [file pcbi.1003915.s008.tif]

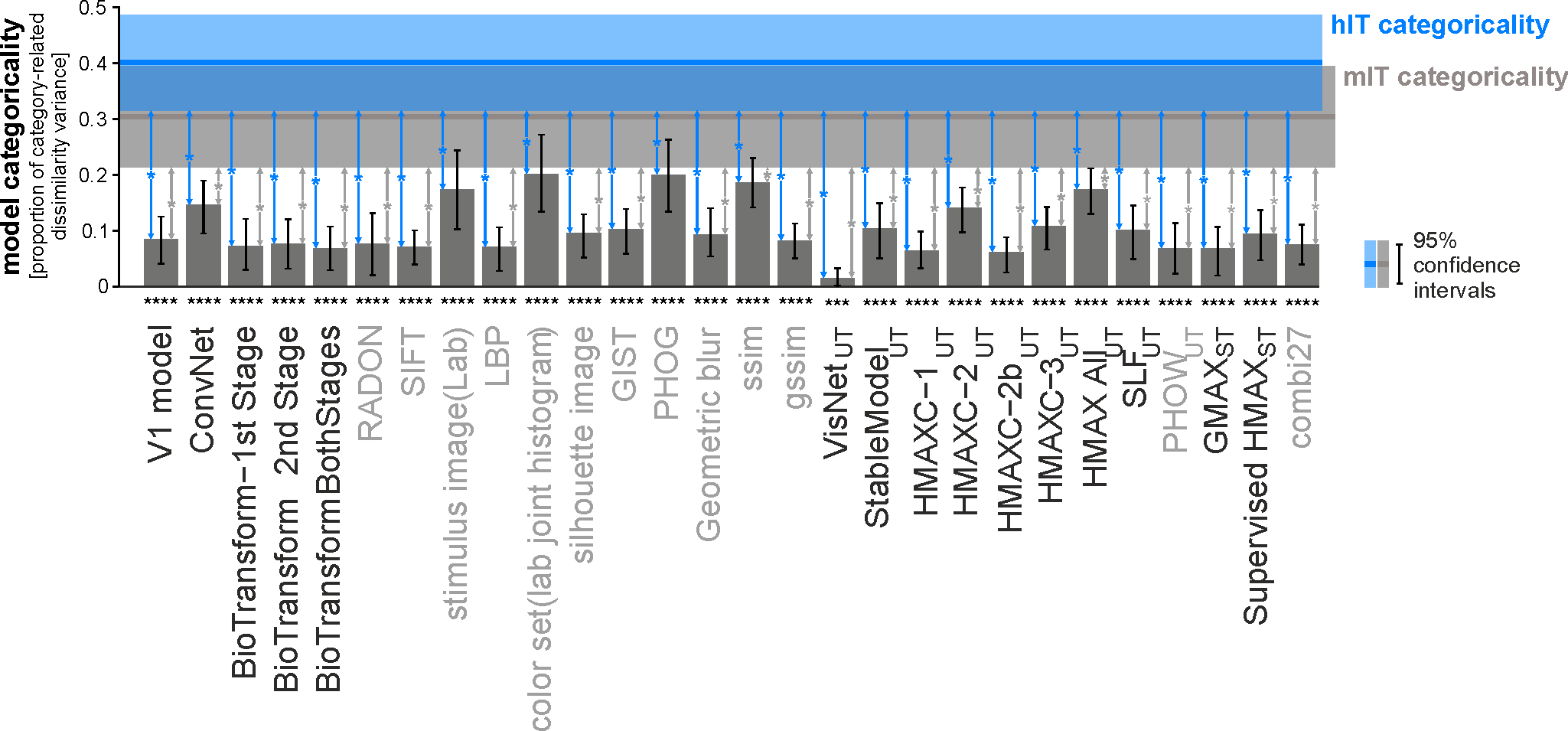

Supplement: Figure S9 — Categoricality in noise-less models compared with the categoricality in IT. Bars show categoricality (measured by the category clustering index, CCI) for each of the not-strongly-supervised models.The category clustering index (CCI) for each model and brain RDM is defined as the proportion of RDM variance explained by the category cluster model (Figure S5), i.e. the squared correlation between the fitted category-cluster model and the RDM it is fitted to. Error bars and shaded regions indicate 95%-confidence intervals. Significant CCIs are indicated by stars underneath the bars (* p<0.05, ** p<0.01, *** p<0.001, **** p<0.0001). Significant differences between the CCI of each model and the hIT/mIT CCI are indicated by blue/gray vertical arrows (p<0.05, Bonferroni-adjusted for 28 tests). The corresponding inferential comparisons for mIT are indicated by gray vertical arrows. The categoricality in hIT is significantly higher than in any of the 28 not-strongly-supervised models. This analysis is based on the noise-less model representations. (TIF) [file pcbi.1003915.s009.tif]

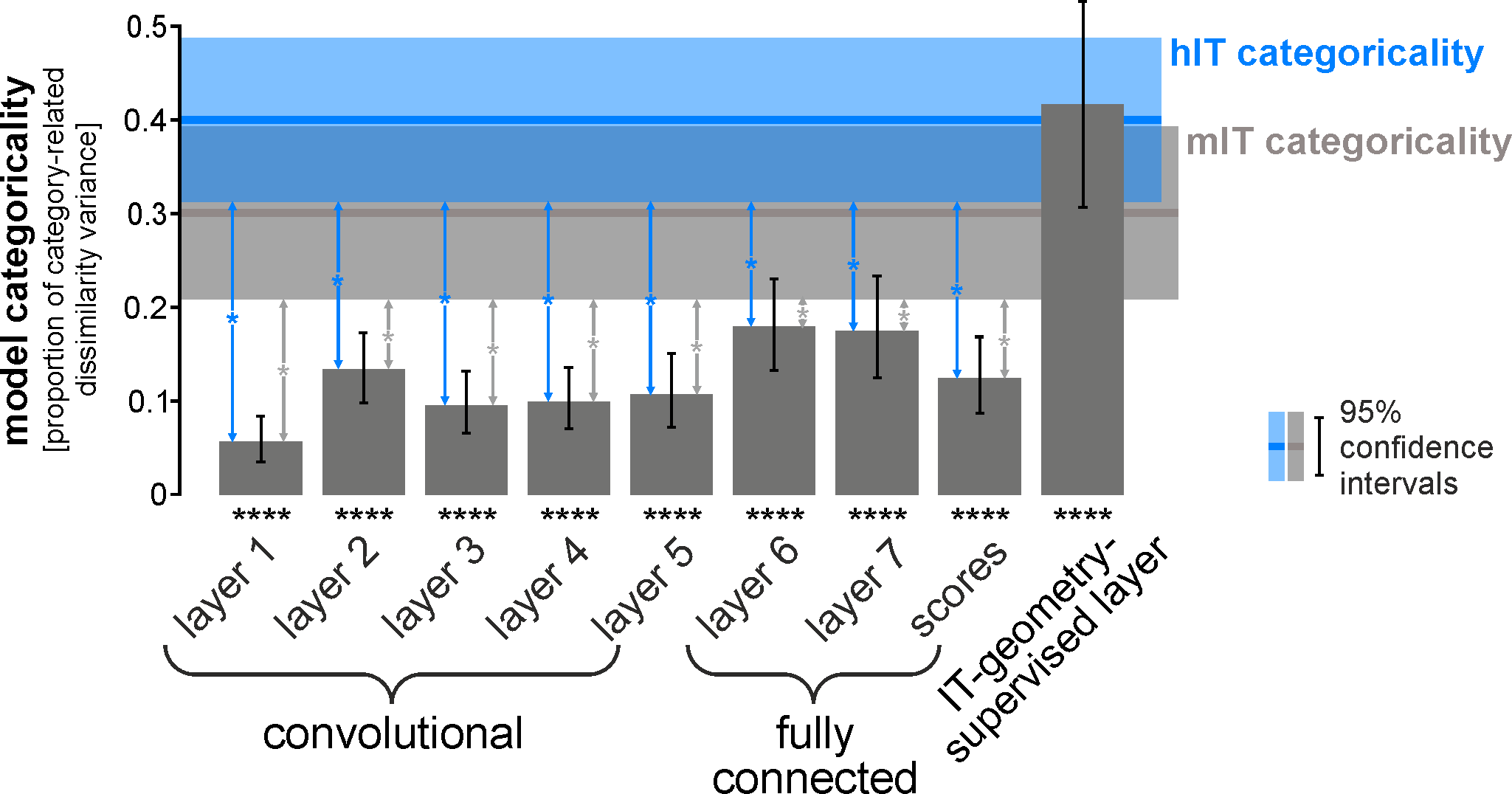

Supplement: Figure S10 — Categoricality in the noise-less representations of the deep convolutional network compared with hIT and mIT. Bars show categoricality (measured by the category clustering index, CCI) for each layer of the deep convolutional network and for the IT-geometry-supervised layer. For conventions and for definition of the CCI, see Figure S9. Error bars and shaded regions indicate 95%-confidence intervals. Significant CCIs are indicated by stars underneath the bars (* p<0.05, ** p<0.01, *** p<0.001, **** p<0.0001). Significant differences between the CCI of each model and the hIT/mIT CCI are indicated by blue/gray vertical arrows (p<0.05, Bonferroni-adjusted for 9 tests). The corresponding inferential comparisons for mIT are indicated by gray vertical arrows. Categoricality is significantly greater in hIT and mIT than in any of the internal layers of the deep convolutional network. However, the IT-geometry-supervised layer (remixed and reweighted) achieves a categoricality similar to IT. This analysis is based on the noise-less model representations. (TIF) [file pcbi.1003915.s010.tif]

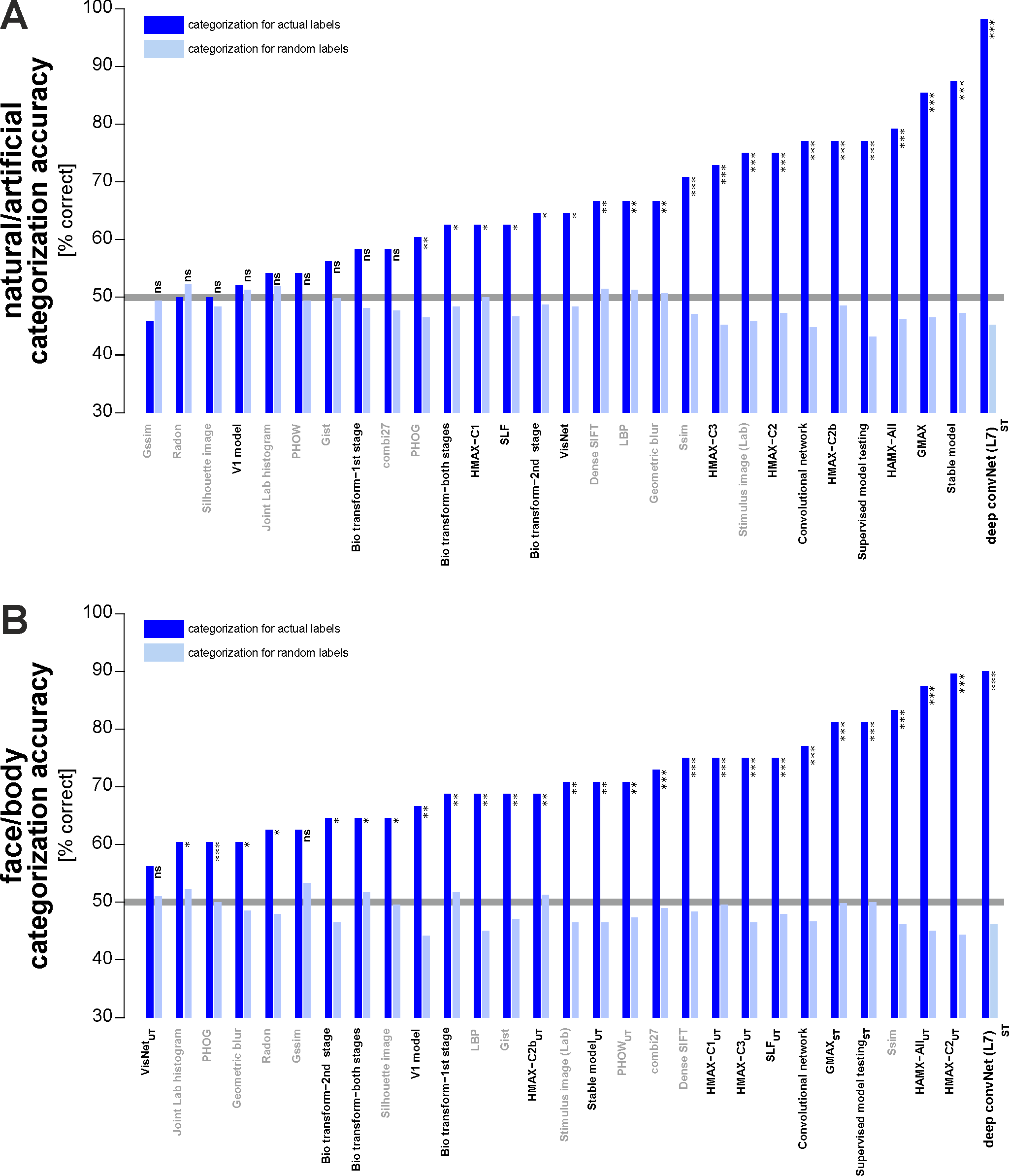

Supplement: Figure S11 — Categorization accuracy of all models for natural/artificial (A) and face/body (B). Each dark blue bar shows the categorization accuracy of a linear SVM applied to one of the computational model representations. Categorization accuracy for each model was estimated by 12-fold crossvalidation on the 96 stimuli. To assess whether categorization accuracy was above chance level, we performed a permutation test, in which we retrained the SVMs on (category-orthogonalized) 10,000 random dichotomies among the stimuli. Light blue bars show the average model categorization accuracy for random label permutations. Categorization performance was significantly greater than chance for most models (ns: not significant, * p<0.05, ** p<0.01, *** p<0.001, **** p<0.0001). (TIF) [file pcbi.1003915.s011.tif]

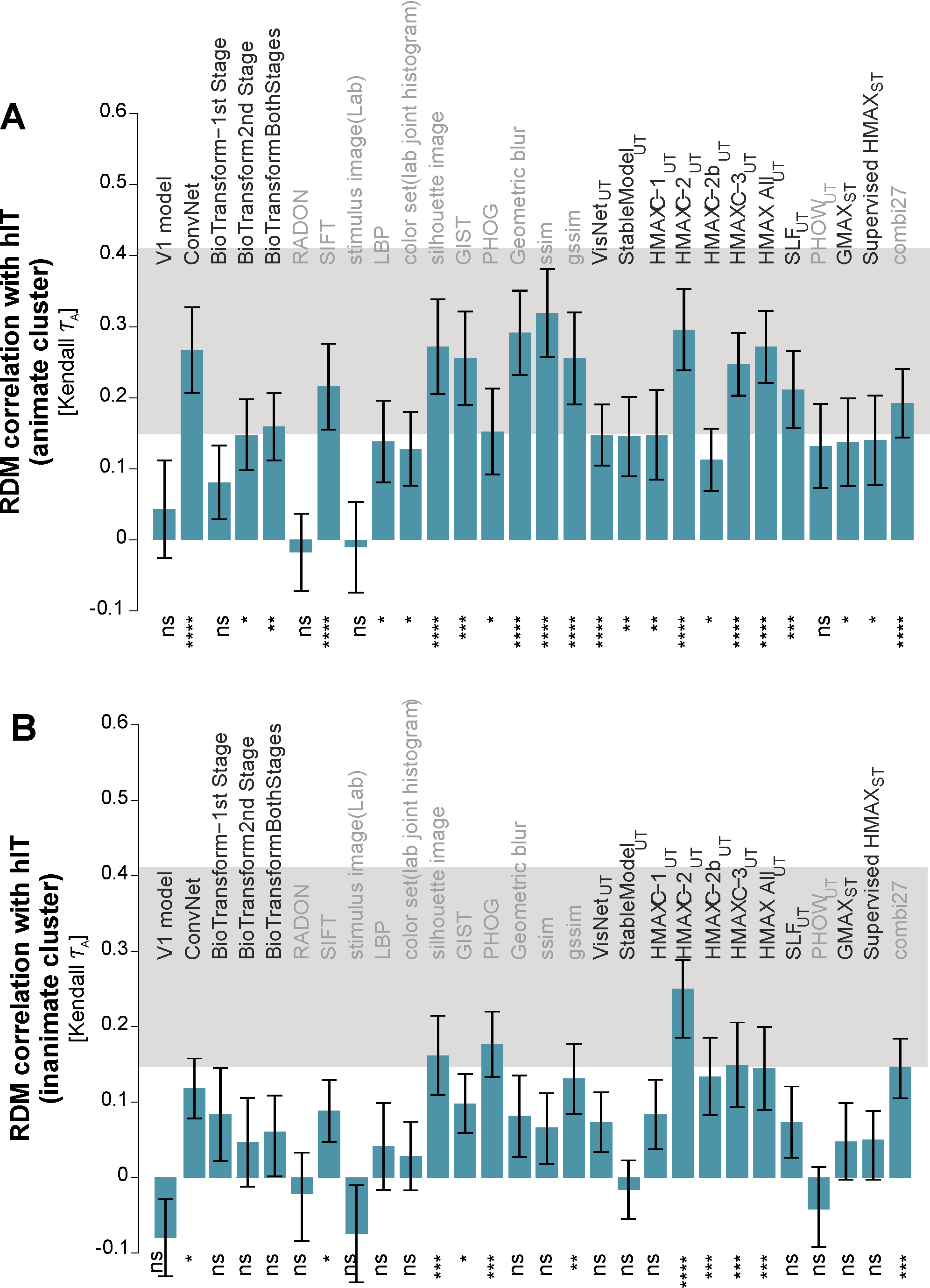

Supplement: Figure S12 — Kendall's τ A RDM correlation of the not-strongly-supervised models with the hIT animate (A) and inanimate (B) sub-clusters. The bars show the Kendall-τA RDM correlations of the not-strongly-supervised models with the hIT RDM for animate images (A), and inanimate images (B). The error bars are standard deviations of the mean estimated by bootstrap resampling. Asterisks across the x-axis show the p-values obtained by a random permutation test based on 10,000 randomizations of the condition labels (ns: not significant, p<0.05: *, p<0.01: **, p<0.001: ***, p<0.0001: ****). The p-values assess the relatedness of different model RDMs with a brain RDM. The grey horizontal rectangles show the noise ceiling. Models with the subscript ‘UT’ are unsupervised trained models, models with the subscript ‘ST’ are supervised trained models, and others without a subscript are untrained models. (TIF) [file pcbi.1003915.s012.tif]

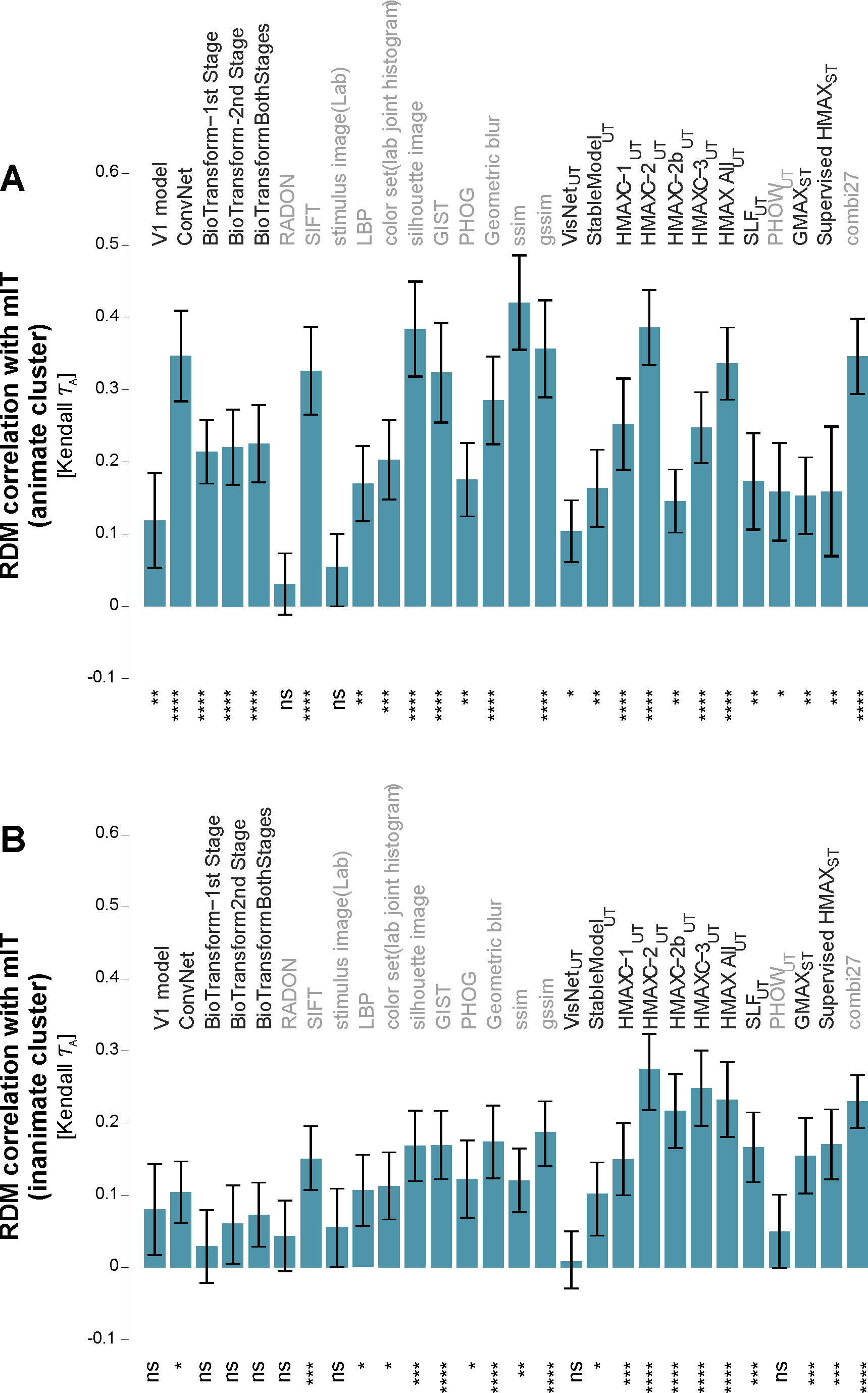

Supplement: Figure S13 — Kendall's τ A RDM correlation of the not-strongly-supervised models with the mIT animate (A) and inanimate (B) sub-clusters. The bars show the Kendall-τA RDM correlations of the not-strongly-supervised models with the mIT RDM for animate images (A), and inanimate images (B). The error bars are standard deviations of the mean estimated by bootstrap resampling. Asterisks across the x-axis show the p-values obtained by a random permutation test based on 10,000 randomizations of the condition labels (ns: not significant, p<0.05: *, p<0.01: **, p<0.001: ***, p<0.0001: ****). The p-values assess the relatedness of different model RDMs with a brain RDM. Models with the subscript ‘UT’ are unsupervised trained models, models with the subscript ‘ST’ are supervised trained models, and others without a subscript are untrained models. (TIF) [file pcbi.1003915.s013.tif]
