# Supplementary material for: Deep Supervised, but Not Unsupervised, Models May Explain IT Cortical Representation
Source: PLoS Comput Biol. 2014 Nov 6;10(11):e1003915. doi: 10.1371/journal.pcbi.1003915 (PMC4222664; doi:10.1371/journal.pcbi.1003915)
Supplement: Text S1 — Models better explain the representation of the animate objects than the inanimate objects in IT. (DOCX) [file pcbi.1003915.s014.docx]

Text S1. Models better explain the representation of the animate objects than the inanimate objects in IT. Figures S12 and S13 show within-category (i.e. animate and inanimate) correlations of model RDMs with the hIT and mIT RDMs. The models better explained the IT representational geometry for the animate category, compared to the inanimate category. For the animate category (Figure S12A) many object-vision models (e.g. ssim, convNet, gist, HMAX, geometric blur, silhouette image) are within the range of the noise ceiling and a few of them even close to the upper bound of the noise ceiling (e.g. ssim, HMAX-C2). This suggests that for animate objects the models can reasonably well predict the IT representational geometry. However for the inanimate objects, RDM correlation of the models with IT (Figure S12B, and S13B) is systematically lower than the animate objects. This further shows that when visual similarity among the stimuli is higher (i.e. animate cluster), the unsupervised models perform reasonably well in explaining the IT representation. In contrast if the object category is more driven by semantic rather than visual similarity (i.e. inanimate cluster) then the unsupervised models fail to fully explain the IT representational geometry. This also emphasizes the importance of stimulus selection for exploring the IT representation. If in this study, for each semantic category, we were only selecting stimuli with high within-category visual similarity then we would have overestimated the ability of the unsupervised object-vision models in explaining the IT representational geometry.
